# Supplementary material for: Cardiopulmonary, metabolic, and perceptual responses during exercise in Myalgic Encephalomyelitis/Chronic Fatigue Syndrome (ME/CFS): A Multi-site Clinical Assessment of ME/CFS (MCAM) sub-study
Source: PLoS One. 2022 Mar 15;17(3):e0265315. doi: 10.1371/journal.pone.0265315 (PMC8923458; doi:10.1371/journal.pone.0265315)
Supplement: S1 Data — (PDF) [file pone.0265315.s001.pdf]

Group Statistics

	Group	N	Mean	Std. Deviation	Std. Error Mean
Sys_BP_rest	ME_CFS	168	121.9167	14.17530	1.09365
	Control	151	121.1921	15.77454	1.28371
Dia_BP_rest	ME_CFS	168	79.7262	9.87751	.76207
	Control	150	76.4933	10.58031	.86388
HR_Rest	ME_CFS	173	67.7861	11.61121	.88278
	Control	157	62.2548	10.04790	.80191

Independent Samples Effect Sizes

				95% Confidence Interval	
		Standardizer[a]	Point Estimate	Lower	Upper
Sys_BP_rest	Cohen's d	14.95337	.048	-.171	.268
	Hedges' correction	14.98887	.048	-.171	.268
	Glass's delta	15.77454	.046	-.174	.266
Dia_BP_rest	Cohen's d	10.21492	.316	.095	.538
	Hedges' correction	10.23925	.316	.094	.537
	Glass's delta	10.58031	.306	.082	.528
HR_Rest	Cohen's d	10.89569	.508	.288	.727
	Hedges' correction	10.92069	.507	.287	.725
	Glass's delta	10.04790	.550	.325	.774

a. The denominator used in estimating the effect sizes.
Cohen's d uses the pooled standard deviation.
Hedges' correction uses the pooled standard deviation, plus a correction factor.
Glass's delta uses the sample standard deviation of the control group.

Group Statistics

	Group	N	Mean	Std. Deviation	Std. Error Mean
VO2_mls_Vslope	ME_CFS	145	949.4211	399.95282	33.21427
	Control	149	1112.1450	521.76511	42.74466
RER_Vslope	ME_CFS	145	.8420333188	.0718900952	.0059701475
	Control	149	.8608479601	.0786819291	.0064458748
VE_stpd_Vslope	ME_CFS	145	18.78701969	6.987891209	.5803127786
	Control	149	22.74266928	9.879422495	.8093538264
RR_Vslope	ME_CFS	145	19.77020860	5.347457713	.4440821918
	Control	149	22.15507991	4.738690886	.3882086835
HR_Vslope	ME_CFS	145	104.1279656	16.88768938	1.402446268
	Control	149	108.0259939	19.40182943	1.589459799
O2pulse_Vslope	ME_CFS	145	9.1295	3.49228	.29002
	Control	149	10.2986	4.18966	.34323
VE_VO2_Vslope	ME_CFS	145	25.34335979	5.065996714	.4207081280
	Control	149	23.58365964	3.327601928	.2726077718
VE_VCO2_Vslope	ME_CFS	145	30.27888535	6.533750197	.5425984203
	Control	149	27.68813762	3.514064028	.2878833423
Watts_Vslope	ME_CFS	145	57.87482276	28.14768020	2.337537609
	Control	149	74.75633904	36.26971806	2.971331079
TV.thres	ME_CFS	136	1.030384472	.3976727791	.0341001595
	Control	148	1.041061650	.4052746551	.0333133714
CI.thres	ME_CFS	139	.9352228508	.1492790875	.0126616869
	Control	139	.9748869027	.1704869226	.0144605120

Independent Samples Effect Sizes

		Standardizer[a]	Point Estimate	95% Confidence Interval Lower	95% Confidence Interval Upper
VO2_mls_Vslope	Cohen's d	465.69256	-.349	-.580	-.119
	Hedges' correction	466.89298	-.349	-.578	-.118
	Glass's delta	521.76511	-.312	-.543	-.080
RER_Vslope	Cohen's d	.0754090207	-.250	-.479	-.020
	Hedges' correction	.0756034017	-.249	-.478	-.020
	Glass's delta	.0786819291	-.239	-.469	-.008
VE_stpd_Vslope	Cohen's d	8.576180006	-.461	-.693	-.229
	Hedges' correction	8.598286730	-.460	-.691	-.229
	Glass's delta	9.879422495	-.400	-.633	-.167
RR_Vslope	Cohen's d	5.048087963	-.472	-.704	-.240
	Hedges' correction	5.061100364	-.471	-.702	-.240
	Glass's delta	4.738690886	-.503	-.738	-.267
HR_Vslope	Cohen's d	18.20542308	-.214	-.443	.015
	Hedges' correction	18.25235100	-.214	-.442	.015
	Glass's delta	19.40182943	-.201	-.430	.029
O2pulse_Vslope	Cohen's d	3.86152	-.303	-.532	-.073
	Hedges' correction	3.87147	-.302	-.531	-.072
	Glass's delta	4.18966	-.279	-.509	-.048
VE_VO2_Vslope	Cohen's d	4.274188535	.412	.180	.642
	Hedges' correction	4.285206063	.411	.180	.641
	Glass's delta	3.327601928	.529	.292	.764
VE_VCO2_Vslope	Cohen's d	5.226035980	.496	.263	.727
	Hedges' correction	5.239507075	.494	.263	.726
	Glass's delta	3.514064028	.737	.493	.980
Watts_Vslope	Cohen's d	32.51885207	-.519	-.751	-.286
	Hedges' correction	32.60267555	-.518	-.749	-.285
	Glass's delta	36.26971806	-.465	-.699	-.230
TV.thres	Cohen's d	.4016534116	-.027	-.259	.206
	Hedges' correction	.4027255993	-.027	-.259	.206
	Glass's delta	.4052746551	-.026	-.259	.207
CI.thres	Cohen's d	.1602342609	-.248	-.483	-.011
	Hedges' correction	.1606713297	-.247	-.482	-.011
	Glass's delta	.1704869226	-.233	-.469	.004

a. The denominator used in estimating the effect sizes.
Cohen's d uses the pooled standard deviation.
Hedges' correction uses the pooled standard deviation, plus a correction factor.
Glass's delta uses the sample standard deviation of the control group.

Group Statistics

	Group	N	Mean	Std. Deviation	Std. Error Mean
VO2_mls_Vslope	ME_CFS	82	1019.5054	398.86799	44.04760
	Control	88	968.5196	407.14286	43.40157
RER_Vslope	ME_CFS	82	.8459461487	.0743487310	.0082104438
	Control	88	.8703203594	.0804110313	.0085718447
VE_stpd_Vslope	ME_CFS	82	20.03647099	7.152692009	.7898826941
	Control	88	20.60204013	8.399369532	.8953758006
RR_Vslope	ME_CFS	82	19.20516470	4.931505493	.5445936774
	Control	88	21.47959813	5.074313979	.5409236877
HR_Vslope	ME_CFS	82	105.2137708	15.92125363	1.758208336
	Control	88	105.4732807	18.53705393	1.976056589
O2pulse_Vslope	ME_CFS	82	9.6712	3.52132	.38886
	Control	88	9.3708	4.10122	.43719
VE_VO2_Vslope	ME_CFS	82	24.62245478	4.524627679	.4996615395
	Control	88	23.79086324	3.837710298	.4091012924
VE_VCO2_Vslope	ME_CFS	82	29.31935950	6.024112555	.6652519427
	Control	88	27.72007284	3.424851162	.3650903606
Watts_Vslope	ME_CFS	82	62.80630290	28.96418546	3.198559202
	Control	88	65.92884732	28.78985145	3.069008476
TV.thres	ME_CFS	80	1.138448866	.4377541945	.0489424068
	Control	88	.9834469471	.3678314851	.0392109681
CI.thres	ME_CFS	81	.9342820278	.1283567514	.0142618613
	Control	84	.9842415085	.2059497645	.0224709616

Independent Samples Effect Sizes

		Standardizer[a]	Point Estimate	95% Confidence Interval	
				Lower	Upper
VO2_mls_Vslope	Cohen's d	403.17439	.126	-.175	.427
	Hedges' correction	404.98551	.126	-.174	.425
	Glass's delta	407.14286	.125	-.177	.426
RER_Vslope	Cohen's d	.0775473239	-.314	-.617	-.011
	Hedges' correction	.0778956774	-.313	-.614	-.011
	Glass's delta	.0804110313	-.303	-.606	.002
VE_stpd_Vslope	Cohen's d	7.823134109	-.072	-.373	.229
	Hedges' correction	7.858276723	-.072	-.371	.228
	Glass's delta	8.399369532	-.067	-.368	.234
RR_Vslope	Cohen's d	5.005968513	-.454	-.758	-.149
	Hedges' correction	5.028456024	-.452	-.755	-.148
	Glass's delta	5.074313979	-.448	-.755	-.139
HR_Vslope	Cohen's d	17.32523926	-.015	-.316	.286
	Hedges' correction	17.40306666	-.015	-.314	.285
	Glass's delta	18.53705393	-.014	-.315	.287
O2pulse_Vslope	Cohen's d	3.83259	.078	-.223	.379
	Hedges' correction	3.84981	.078	-.222	.378
	Glass's delta	4.10122	.073	-.228	.374
VE_VO2_Vslope	Cohen's d	4.183008768	.199	-.103	.500
	Hedges' correction	4.201799429	.198	-.103	.498
	Glass's delta	3.837710298	.217	-.086	.519
VE_VCO2_Vslope	Cohen's d	4.855017149	.329	.026	.632
	Hedges' correction	4.876826566	.328	.026	.629
	Glass's delta	3.424851162	.467	.157	.774
Watts_Vslope	Cohen's d	28.87403675	-.108	-.409	.193
	Hedges' correction	29.00374297	-.108	-.407	.192
	Glass's delta	28.78985145	-.108	-.409	.193
TV.thres	Cohen's d	.4026251970	.385	.079	.690
	Hedges' correction	.4044557743	.383	.078	.687
	Glass's delta	.3678314851	.421	.111	.729
CI.thres	Cohen's d	.1722907161	-.290	-.596	.017
	Hedges' correction	.1730885637	-.289	-.594	.017
	Glass's delta	.2059497645	-.243	-.549	.066

a. The denominator used in estimating the effect sizes.
Cohen's d uses the pooled standard deviation.
Hedges' correction uses the pooled standard deviation, plus a correction factor.
Glass's delta uses the sample standard deviation of the control group.

Group Statistics

	Group	N	Mean	Std. Deviation	Std. Error Mean
VO2_mls_peak	ME_CFS	145	1848.4236	721.87945	59.94883
	Control	149	2161.4352	771.76925	63.22580
VCO2_mls_peak	ME_CFS	145	2142.2745	774.58476	64.32576
	Control	149	2465.8805	785.17985	64.32444
VO2_kg_PEAK	ME_CFS	145	24.25695377	8.883822755	.7377613236
	Control	149	30.36633508	10.91044938	.8938188400
RER_PEAK	ME_CFS	145	1.173797914	.1058264724	.0087884102
	Control	149	1.158188110	.0887032009	.0072668494
VE_STPD_PEAK	ME_CFS	145	55.27159070	20.96697466	1.741212472
	Control	149	64.25596239	20.87235139	1.709929651
RR_PEAK	ME_CFS	145	34.03919113	9.591261982	.7965109537
	Control	149	39.37180802	8.816799997	.7223003994
HR_PEAK	ME_CFS	145	158.4482759	19.38926215	1.610190579
	Control	149	166.6971477	17.16318723	1.406063084
O2pulse_peak	ME_CFS	145	11.6337	4.15839	.34534
	Control	149	12.9858	4.64214	.38030
VE_VO2_PEAK	ME_CFS	145	38.13189594	8.769810733	.7282931406
	Control	149	34.48800663	5.767481658	.4724905075
VE_VCO2_PEAK	ME_CFS	145	32.51144122	6.950667116	.5772214860
	Control	149	29.81712186	4.695410585	.3846630231
Time to Peak (sec)	ME_CFS	145	673.07	184.961	15.360
	Control	149	751.98	214.159	17.545
Watts_PEAK	ME_CFS	145	141.99	43.455	3.609
	Control	149	165.62	51.088	4.185
TV.max	ME_CFS	137	1.829767431	.5775453042	.0493430253
	Control	145	1.754155856	.5837015583	.0484737760
CI.max	ME_CFS	141	1.328112520	1.003889273	.0845427280
	Control	139	1.319171498	.3465997707	.0293982089

Independent Samples Effect Sizes

		Standardizer[a]	Point Estimate	95% Confidence Interval Lower	95% Confidence Interval Upper
VO2_mls_peak	Cohen's d	747.58227	-.419	-.649	-.187
	Hedges' correction	749.50931	-.418	-.648	-.187
	Glass's delta	771.76925	-.406	-.638	-.172
VCO2_mls_peak	Cohen's d	779.97286	-.415	-.646	-.183
	Hedges' correction	781.98339	-.414	-.644	-.183
	Glass's delta	785.17985	-.412	-.645	-.178
VO2_kg_PEAK	Cohen's d	9.962673867	-.613	-.847	-.379
	Hedges' correction	9.988354541	-.612	-.845	-.378
	Glass's delta	10.91044938	-.560	-.796	-.322
RER_PEAK	Cohen's d	.0975240231	.160	-.069	.389
	Hedges' correction	.0977754097	.160	-.069	.388
	Glass's delta	.0887032009	.176	-.054	.405
VE_STPD_PEAK	Cohen's d	20.91906841	-.429	-.660	-.198
	Hedges' correction	20.97299126	-.428	-.659	-.197
	Glass's delta	20.87235139	-.430	-.664	-.196
RR_PEAK	Cohen's d	9.206871787	-.579	-.812	-.345
	Hedges' correction	9.230604239	-.578	-.810	-.344
	Glass's delta	8.816799997	-.605	-.843	-.365
HR_PEAK	Cohen's d	18.29486056	-.451	-.682	-.219
	Hedges' correction	18.34201902	-.450	-.680	-.218
	Glass's delta	17.16318723	-.481	-.715	-.245
O2pulse_peak	Cohen's d	4.41021	-.307	-.536	-.076
	Hedges' correction	4.42158	-.306	-.535	-.076
	Glass's delta	4.64214	-.291	-.522	-.060
VE_VO2_PEAK	Cohen's d	7.401876052	.492	.260	.724
	Hedges' correction	7.420955786	.491	.259	.722
	Glass's delta	5.767481658	.632	.391	.871
VE_VCO2_PEAK	Cohen's d	5.916031646	.455	.223	.687
	Hedges' correction	5.931281335	.454	.223	.685
	Glass's delta	4.695410585	.574	.335	.811
Time to Peak (sec)	Cohen's d	200.293	-.394	-.625	-.163
	Hedges' correction	200.809	-.393	-.623	-.162
	Glass's delta	214.159	-.368	-.600	-.135

Independent Samples Effect Sizes

		Standardizer[a]	Point Estimate	95% Confidence Interval	
				Lower	Upper
Watts_PEAK	Cohen's d	47.477	-.498	-.730	-.265
	Hedges' correction	47.600	-.497	-.728	-.265
	Glass's delta	51.088	-.463	-.697	-.227
TV.max	Cohen's d	.5807195290	.130	-.104	.364
	Hedges' correction	.5822808363	.130	-.103	.363
	Glass's delta	.5837015583	.130	-.105	.363
CI.max	Cohen's d	.7530973152	.012	-.222	.246
	Hedges' correction	.7551366943	.012	-.222	.245
	Glass's delta	.3465997707	.026	-.209	.260

a. The denominator used in estimating the effect sizes.
Cohen's d uses the pooled standard deviation.
Hedges' correction uses the pooled standard deviation, plus a correction factor.
Glass's delta uses the sample standard deviation of the control group.

Group Statistics

	Group	N	Mean	Std. Deviation	Std. Error Mean
VO2_mls_peak	ME_CFS	82	2007.1298	712.09382	78.63761
	Control	88	1918.3909	698.15245	74.42330
VCO2_mls_peak	ME_CFS	82	2324.0209	771.44781	85.19216
	Control	88	2220.0606	719.13697	76.66026
VO2_kg_PEAK	ME_CFS	82	26.58197507	9.048563936	.9992467242
	Control	88	25.72381149	8.868659115	.9454022382
RER_PEAK	ME_CFS	82	1.170705054	.0974483352	.0107613684
	Control	88	1.172454734	.0883121562	.0094141075
VE_STPD_PEAK	ME_CFS	82	60.05470301	22.59351187	2.495035996
	Control	88	58.21178299	19.93185301	2.124742670
RR_PEAK	ME_CFS	82	33.58076355	10.27810628	1.135026962
	Control	88	38.08620084	9.179425063	.9785299996
HR_PEAK	ME_CFS	82	160.6097561	17.88009176	1.974525820
	Control	88	162.0681818	17.15500360	1.828729528
O2pulse_peak	ME_CFS	82	12.4568	4.04140	.44630
	Control	88	11.8916	4.46290	.47575
VE_VO2_PEAK	ME_CFS	82	37.23842218	8.334519302	.9203936857
	Control	88	34.46150426	5.911930525	.6302139115

Group Statistics

	Group	N	Mean	Std. Deviation	Std. Error Mean
VE_VCO2_PEAK	ME_CFS	82	31.89595870	6.903503980	.7623644798
	Control	88	29.44400339	4.630008875	.4935606045
Time to Peak (sec)	ME_CFS	82	714.73	184.804	20.408
	Control	88	686.60	193.383	20.615
Watts_PEAK	ME_CFS	82	152.45	43.906	4.849
	Control	88	149.39	48.365	5.156
TV.max	ME_CFS	80	2.008627059	.6223936908	.0695857301
	Control	87	1.662997203	.5683751477	.0609362120
CI.max	ME_CFS	81	1.373715728	1.274650591	.1416278435
	Control	84	1.361762741	.3932023840	.0429018973

Independent Samples Effect Sizes

		Standardizer[a]	Point Estimate	95% Confidence Interval	
				Lower	Upper
VO2_mls_peak	Cohen's d	704.90860	.126	-.175	.427
	Hedges' correction	708.07515	.125	-.175	.425
	Glass's delta	698.15245	.127	-.175	.428
VCO2_mls_peak	Cohen's d	744.81707	.140	-.162	.441
	Hedges' correction	748.16289	.139	-.161	.439
	Glass's delta	719.13697	.145	-.157	.446
VO2_kg_PEAK	Cohen's d	8.955850115	.096	-.205	.397
	Hedges' correction	8.996081048	.095	-.204	.395
	Glass's delta	8.868659115	.097	-.205	.398
RER_PEAK	Cohen's d	.0928294209	-.019	-.320	.282
	Hedges' correction	.0932464237	-.019	-.318	.281
	Glass's delta	.0883121562	-.020	-.321	.281
VE_STPD_PEAK	Cohen's d	21.25680026	.087	-.214	.388
	Hedges' correction	21.35228878	.086	-.213	.386
	Glass's delta	19.93185301	.092	-.209	.393
RR_PEAK	Cohen's d	9.724654922	-.463	-.768	-.158
	Hedges' correction	9.768339434	-.461	-.764	-.157
	Glass's delta	9.179425063	-.491	-.799	-.180

Independent Samples Effect Sizes

		Standardizer[a]	Point Estimate	95% Confidence Interval	
				Lower	Upper
HR_PEAK	Cohen's d	17.50834888	-.083	-.384	.218
	Hedges' correction	17.58699883	-.083	-.382	.217
	Glass's delta	17.15500360	-.085	-.386	.216
O2pulse_peak	Cohen's d	4.26488	.133	-.169	.433
	Hedges' correction	4.28404	.132	-.168	.432
	Glass's delta	4.46290	.127	-.175	.428
VE_VO2_PEAK	Cohen's d	7.182705516	.387	.082	.690
	Hedges' correction	7.214971235	.385	.082	.687
	Glass's delta	5.911930525	.470	.160	.777
VE_VCO2_PEAK	Cohen's d	5.837759474	.420	.115	.724
	Hedges' correction	5.863983507	.418	.115	.720
	Glass's delta	4.630008875	.530	.217	.839
Time to Peak (sec)	Cohen's d	189.295	.149	-.153	.450
	Hedges' correction	190.146	.148	-.152	.448
	Glass's delta	193.383	.145	-.157	.447
Watts_PEAK	Cohen's d	46.269	.066	-.235	.367
	Hedges' correction	46.476	.066	-.234	.365
	Glass's delta	48.365	.063	-.238	.364
TV.max	Cohen's d	.5948509624	.581	.270	.890
	Hedges' correction	.5975720096	.578	.269	.886
	Glass's delta	.5683751477	.608	.290	.923
CI.max	Cohen's d	.9360247621	.013	-.292	.318
	Hedges' correction	.9403593263	.013	-.291	.317
	Glass's delta	.3932023840	.030	-.275	.336

a. The denominator used in estimating the effect sizes.
Cohen's d uses the pooled standard deviation.
Hedges' correction uses the pooled standard deviation, plus a correction factor.
Glass's delta uses the sample standard deviation of the control group.
