# Supplementary material for: Cardiopulmonary, metabolic, and perceptual responses during exercise in Myalgic Encephalomyelitis/Chronic Fatigue Syndrome (ME/CFS): A Multi-site Clinical Assessment of ME/CFS (MCAM) sub-study
Source: PLoS One. 2022 Mar 15;17(3):e0265315. doi: 10.1371/journal.pone.0265315 (PMC8923458; doi:10.1371/journal.pone.0265315)
Supplement: S2 Data — (PDF) [file pone.0265315.s002.pdf]

Mixed Model Analyses

Model Dimension[a]

		Number of Levels	Covariance Structure	Number of Parameters	Subject Variables	Number of Subjects
Fixed Effects	Intercept	1		1		
	Group	2		1		
	Time	5		4		
	Age	1		1		
	Group * Time	10		4		
Repeated Effects	Time	5	Heterogeneous First-Order Autoregressive	6	Participant_Number	282
Total		24		17		

a. Dependent Variable: VE.

Type III Tests of Fixed Effects[a]

Source	Numerator df	Denominator df	F	Sig.
Intercept	1	542.556	1205.194	<.001
Group	1	283.939	10.543	.001
Time	4	500.060	542.687	<.001
Age	1	360.912	1.509	.220
Group * Time	4	500.060	5.080	<.001

a. Dependent Variable: VE.

Model Dimension[a]

		Number of Levels	Covariance Structure	Number of Parameters	Subject Variables	Number of Subjects
Fixed Effects	Intercept	1		1		
	Group	2		1		
	Time	5		4		
	Age	1		1		
	Group * Time	10		4		
Repeated Effects	Time	5	Heterogeneous First-Order Autoregressive	6	Participant_Number	282
Total		24		17		

a. Dependent Variable: RR.

Type III Tests of Fixed Effects[a]

Source	Numerator df	Denominator df	F	Sig.
Intercept	1	332.645	925.369	<.001
Group	1	305.854	29.901	<.001
Time	4	639.223	285.458	<.001
Age	1	329.329	2.664	.104
Group * Time	4	639.223	4.201	.002

a. Dependent Variable: RR.

Model Dimension[a]

		Number of Levels	Covariance Structure	Number of Parameters	Subject Variables	Number of Subjects
Fixed Effects	Intercept	1		1		
	Group	2		1		
	Time	5		4		
	Age	1		1		
	Group * Time	10		4		
Repeated Effects	Time	5	Heterogeneous First-Order Autoregressive	6	Participant_Number	282
Total		24		17		

a. Dependent Variable: HR.

Type III Tests of Fixed Effects[a]

Source	Numerator df	Denominator df	F	Sig.
Intercept	1	343.364	3371.743	<.001
Group	1	287.852	.027	.869
Time	4	559.863	615.505	<.001
Age	1	343.013	106.834	<.001
Group * Time	4	559.863	7.123	<.001

a. Dependent Variable: HR.

Model Dimension[a]

		Number of Levels	Covariance Structure	Number of Parameters	Subject Variables	Number of Subjects
Fixed Effects	Intercept	1		1		
	Group	2		1		
	Time	5		4		
	Age	1		1		
	Group * Time	10		4		
Repeated Effects	Time	5	Heterogeneous First-Order Autoregressive	6	Participant_Number	282
Total		24		17		

a. Dependent Variable: VEVO2.

Type III Tests of Fixed Effects[a]

Source	Numerator df	Denominator df	F	Sig.
Intercept	1	293.094	661.408	<.001
Group	1	287.022	11.765	<.001
Time	4	971.873	449.111	<.001
Age	1	291.673	1.711	.192
Group * Time	4	971.873	.555	.695

a. Dependent Variable: VEVO2.

Model Dimension[a]

		Number of Levels	Covariance Structure	Number of Parameters	Subject Variables	Number of Subjects
Fixed Effects	Intercept	1		1		
	Group	2		1		
	Time	5		4		
	Age	1		1		
	Group * Time	10		4		
Repeated Effects	Time	5	Heterogeneous First-Order Autoregressive	6	Participant_Number	282
Total		24		17		

a. Dependent Variable: VEVCO2.

Type III Tests of Fixed Effects[a]

Source	Numerator df	Denominator df	F	Sig.
Intercept	1	291.691	762.016	<.001
Group	1	284.516	8.552	.004
Time	4	825.057	249.329	<.001
Age	1	288.983	7.691	.006
Group * Time	4	825.057	2.529	.039

a. Dependent Variable: VEVCO2.

Model Dimension[a]

		Number of Levels	Covariance Structure	Number of Parameters	Subject Variables	Number of Subjects
Fixed Effects	Intercept	1		1		
	Group	2		1		
	Time	5		4		
	Age	1		1		
	Group * Time	10		4		
Repeated Effects	Time	5	Heterogeneous First-Order Autoregressive	6	Participant_Number	282
Total		24		17		

a. Dependent Variable: CTI.

Type III Tests of Fixed Effects[a]

Source	Numerator df	Denominator df	F	Sig.
Intercept	1	323.930	1587.677	<.001
Group	1	302.914	6.625	.011
Time	4	764.466	15.511	<.001
Age	1	323.898	.796	.373
Group * Time	4	764.471	.217	.929

a. Dependent Variable: CTI.

Model Dimension[a]

		Number of Levels	Covariance Structure	Number of Parameters	Subject Variables	Number of Subjects
Fixed Effects	Intercept	1		1		
	Group	2		1		
	Time	5		4		
	Age	1		1		
	Group * Time	10		4		
Repeated Effects	Time	5	Heterogeneous First-Order Autoregressive	6	Participant_Number	282
Total		24		17		

a. Dependent Variable: VO2/HR.

Type III Tests of Fixed Effects[a]

Source	Numerator df	Denominator df	F	Sig.
Intercept	1	282.334	199.764	<.001
Group	1	280.972	3.122	.078
Time	4	955.884	259.143	<.001
Age	1	281.290	.035	.852
Group * Time	4	955.885	2.227	.064

a. Dependent Variable: VO2/HR.

Model Dimension[a]

		Number of Levels	Covariance Structure	Number of Parameters	Subject Variables	Number of Subjects
Fixed Effects	Intercept	1		1		
	Group	2		1		
	Time	5		4		
	Age	1		1		
	Group * Time	10		4		
Repeated Effects	Time	5	Heterogeneous First-Order Autoregressive	6	Participant_Number	282
Total		24		17		

a. Dependent Variable: TV.stpd.

Type III Tests of Fixed Effects[a]

Source	Numerator df	Denominator df	F	Sig.
Intercept	1	277.859	416.483	<.001
Group	1	286.481	1.735	.189
Time	4	676.053	368.473	<.001
Age	1	251.730	.570	.451
Group * Time	4	676.053	.430	.787

a. Dependent Variable: TV.stpd.

Model Dimension[a]

		Number of Levels	Covariance Structure	Number of Parameters	Subject Variables	Number of Subjects
Fixed Effects	Intercept	1		1		
	Group	2		1		
	Time	5		4		
	Age	1		1		
	Group * Time	10		4		
Repeated Effects	Time	5	Heterogeneous First-Order Autoregressive	6	Participant_Number	282
Total		24		17		

a. Dependent Variable: RPE.

Type III Tests of Fixed Effects[a]

Source	Numerator df	Denominator df	F	Sig.
Intercept	1	359.016	2368.608	<.001
Group	1	317.642	15.019	<.001
Time	4	827.091	950.109	<.001
Age	1	345.972	2.926	.088
Group * Time	4	827.064	.784	.536

a. Dependent Variable: RPE.

Model Dimension[a]

		Number of Levels	Covariance Structure	Number of Parameters	Subject Variables	Number of Subjects
Fixed Effects	Intercept	1		1		
	Group	2		1		
	Time	5		4		
	Age	1		1		
	Group * Time	10		4		
Repeated Effects	Time	5	Heterogeneous First-Order Autoregressive	6	Participant_Number	160
Total		24		17		

a. Dependent Variable: VE.

Type III Tests of Fixed Effects[a]

Source	Numerator df	Denominator df	F	Sig.
Intercept	1	307.944	585.283	<.001
Group	1	159.219	1.047	.308
Time	4	284.879	317.303	<.001
Age	1	208.213	.708	.401
Group * Time	4	284.879	2.188	.070

a. Dependent Variable: VE.

Model Dimension[a]

		Number of Levels	Covariance Structure	Number of Parameters	Subject Variables	Number of Subjects
Fixed Effects	Intercept	1		1		
	Group	2		1		
	Time	5		4		
	Age	1		1		
	Group * Time	10		4		
Repeated Effects	Time	5	Heterogeneous First-Order Autoregressive	6	Participant_Number	160
Total		24		17		

a. Dependent Variable: RR.

Type III Tests of Fixed Effects[a]

Source	Numerator df	Denominator df	F	Sig.
Intercept	1	189.704	417.120	<.001
Group	1	171.221	21.842	<.001
Time	4	354.484	135.169	<.001
Age	1	188.475	2.726	.100
Group * Time	4	354.484	2.040	.088

a. Dependent Variable: RR.

Model Dimension[a]

		Number of Levels	Covariance Structure	Number of Parameters	Subject Variables	Number of Subjects
Fixed Effects	Intercept	1		1		
	Group	2		1		
	Time	5		4		
	Age	1		1		
	Group * Time	10		4		
Repeated Effects	Time	5	Heterogeneous First-Order Autoregressive	6	Participant_Number	160
Total		24		17		

a. Dependent Variable: HR.

Type III Tests of Fixed Effects[a]

Source	Numerator df	Denominator df	F	Sig.
Intercept	1	195.195	1712.983	<.001
Group	1	165.825	.195	.659
Time	4	348.471	423.900	<.001
Age	1	194.573	43.488	<.001
Group * Time	4	348.471	1.738	.141

a. Dependent Variable: HR.

Model Dimension[a]

		Number of Levels	Covariance Structure	Number of Parameters	Subject Variables	Number of Subjects
Fixed Effects	Intercept	1		1		
	Group	2		1		
	Time	5		4		
	Age	1		1		
	Group * Time	10		4		
Repeated Effects	Time	5	Heterogeneous First-Order Autoregressive	6	Participant_Number	160
Total		24		17		

a. Dependent Variable: VEVO2.

Type III Tests of Fixed Effects[a]

Source	Numerator df	Denominator df	F	Sig.
Intercept	1	165.093	300.035	<.001
Group	1	161.452	2.292	.132
Time	4	545.312	265.264	<.001
Age	1	164.589	.580	.448
Group * Time	4	545.312	2.971	.019

a. Dependent Variable: VEVO2.

Model Dimension[a]

		Number of Levels	Covariance Structure	Number of Parameters	Subject Variables	Number of Subjects
Fixed Effects	Intercept	1		1		
	Group	2		1		
	Time	5		4		
	Age	1		1		
	Group * Time	10		4		
Repeated Effects	Time	5	Heterogeneous First-Order Autoregressive	6	Participant_Number	160
Total		24		17		

a. Dependent Variable: VEVCO2.

Type III Tests of Fixed Effects[a]

Source	Numerator df	Denominator df	F	Sig.
Intercept	1	165.687	349.417	<.001
Group	1	159.446	2.822	.095
Time	4	455.379	160.342	<.001
Age	1	164.022	4.183	.042
Group * Time	4	455.379	2.862	.023

a. Dependent Variable: VEVCO2.

Model Dimension[a]

		Number of Levels	Covariance Structure	Number of Parameters	Subject Variables	Number of Subjects
Fixed Effects	Intercept	1		1		
	Group	2		1		
	Time	5		4		
	Age	1		1		
	Group * Time	10		4		
Repeated Effects	Time	5	Heterogeneous First-Order Autoregressive	6	Participant_Number	160
Total		24		17		

a. Dependent Variable: CTI.

Type III Tests of Fixed Effects[a]

Source	Numerator df	Denominator df	F	Sig.
Intercept	1	185.684	706.865	<.001
Group	1	169.042	3.169	.077
Time	4	445.681	13.364	<.001
Age	1	185.546	.185	.667
Group * Time	4	445.687	.714	.583

a. Dependent Variable: CTI.

Model Dimension[a]

		Number of Levels	Covariance Structure	Number of Parameters	Subject Variables	Number of Subjects
Fixed Effects	Intercept	1		1		
	Group	2		1		
	Time	5		4		
	Age	1		1		
	Group * Time	10		4		
Repeated Effects	Time	5	Heterogeneous First-Order Autoregressive	6	Participant_Number	160
Total		24		17		

a. Dependent Variable: VO2/HR.

Type III Tests of Fixed Effects[a]

Source	Numerator df	Denominator df	F	Sig.
Intercept	1	160.452	119.548	<.001
Group	1	156.923	2.289	.132
Time	4	556.668	151.726	<.001
Age	1	159.633	1.536	.217
Group * Time	4	556.666	.815	.516

a. Dependent Variable: VO2/HR.

Model Dimension[a]

		Number of Levels	Covariance Structure	Number of Parameters	Subject Variables	Number of Subjects
Fixed Effects	Intercept	1		1		
	Group	2		1		
	Time	5		4		
	Age	1		1		
	Group * Time	10		4		
Repeated Effects	Time	5	Heterogeneous First-Order Autoregressive	6	Participant_Number	160
Total		24		17		

a. Dependent Variable: TV.stpd.

Type III Tests of Fixed Effects[a]

Source	Numerator df	Denominator df	F	Sig.
Intercept	1	155.268	194.075	<.001
Group	1	159.871	21.902	<.001
Time	4	372.003	196.863	<.001
Age	1	145.694	.194	.660
Group * Time	4	372.003	1.058	.377

a. Dependent Variable: TV.stpd.

Model Dimension[a]

		Number of Levels	Covariance Structure	Number of Parameters	Subject Variables	Number of Subjects
Fixed Effects	Intercept	1		1		
	Group	2		1		
	Time	5		4		
	Age	1		1		
	Group * Time	10		4		
Repeated Effects	Time	5	Heterogeneous First-Order Autoregressive	6	Participant_Number	160
Total		24		17		

a. Dependent Variable: RPE.

Type III Tests of Fixed Effects[a]

Source	Numerator df	Denominator df	F	Sig.
Intercept	1	196.611	1319.978	<.001
Group	1	174.653	14.965	<.001
Time	4	477.164	648.354	<.001
Age	1	184.644	4.525	.035
Group * Time	4	477.132	1.166	.325

a. Dependent Variable: RPE.
