# Supplementary material for: Cardiopulmonary, metabolic, and perceptual responses during exercise in Myalgic Encephalomyelitis/Chronic Fatigue Syndrome (ME/CFS): A Multi-site Clinical Assessment of ME/CFS (MCAM) sub-study
Source: PLoS One. 2022 Mar 15;17(3):e0265315. doi: 10.1371/journal.pone.0265315 (PMC8923458; doi:10.1371/journal.pone.0265315)
Supplement: S3 Data — (PDF) [file pone.0265315.s003.pdf]

Group Statistics

	Group	N	Mean	Std. Deviation	Std. Error Mean
VE.stpd._0.2	ME_CFS	178	14.9786	4.21728	.31610
	Control	169	15.5313	5.05598	.38892
VE.stpd._0.4	ME_CFS	178	20.4193	6.28239	.47088
	Control	169	21.9077	7.09917	.54609
VE.stpd._0.6	ME_CFS	178	28.1327	9.07458	.68017
	Control	169	31.5303	10.00339	.76949
VE.stpd._0.8	ME_CFS	178	38.8422	13.07096	.97971
	Control	169	44.4261	14.17192	1.09015
VE.stpd._1	ME_CFS	178	54.7139	21.39706	1.60378
	Control	169	63.0037	21.15152	1.62704

Independent Samples Effect Sizes

		Standardizer[a]	Point Estimate	95% Confidence Interval	
				Lower	Upper
VE.stpd._0.2	Cohen's d	4.64464	-.119	-.330	.092
	Hedges' correction	4.65477	-.119	-.329	.092
	Glass's delta	5.05598	-.109	-.320	.102
VE.stpd._0.4	Cohen's d	6.69259	-.222	-.433	-.011
	Hedges' correction	6.70718	-.222	-.432	-.011
	Glass's delta	7.09917	-.210	-.421	.002
VE.stpd._0.6	Cohen's d	9.53817	-.356	-.568	-.144
	Hedges' correction	9.55897	-.355	-.567	-.143
	Glass's delta	10.00339	-.340	-.553	-.126
VE.stpd._0.8	Cohen's d	13.61820	-.410	-.622	-.197
	Hedges' correction	13.64790	-.409	-.621	-.197
	Glass's delta	14.17192	-.394	-.608	-.179
VE.stpd._1	Cohen's d	21.27785	-.390	-.602	-.177
	Hedges' correction	21.32424	-.389	-.601	-.176
	Glass's delta	21.15152	-.392	-.606	-.177

a. The denominator used in estimating the effect sizes.
Cohen's d uses the pooled standard deviation.
Hedges' correction uses the pooled standard deviation, plus a correction factor.
Glass's delta uses the sample standard deviation of the control group.

Group Statistics

	Group	N	Mean	Std. Deviation	Std. Error Mean
VE.stpd._0.2	ME_CFS	99	15.2828	3.91962	.39394
	Control	99	14.2414	5.18691	.52130
VE.stpd._0.4	ME_CFS	99	21.1762	6.09012	.61208
	Control	99	19.9962	7.14463	.71806
VE.stpd._0.6	ME_CFS	99	29.2385	9.19982	.92462
	Control	99	28.9429	9.99454	1.00449
VE.stpd._0.8	ME_CFS	99	40.3742	13.30800	1.33750
	Control	99	40.6516	13.83140	1.39011
VE.stpd._1	ME_CFS	99	57.0562	22.74479	2.28594
	Control	99	56.2966	20.23427	2.03362

Independent Samples Effect Sizes

		Standardizer[a]	Point Estimate	95% Confidence Interval	
				Lower	Upper
VE.stpd._0.2	Cohen's d	4.59714	.227	-.053	.506
	Hedges' correction	4.61483	.226	-.053	.504
	Glass's delta	5.18691	.201	-.080	.480
VE.stpd._0.4	Cohen's d	6.63835	.178	-.102	.457
	Hedges' correction	6.66389	.177	-.101	.455
	Glass's delta	7.14463	.165	-.115	.444
VE.stpd._0.6	Cohen's d	9.60540	.031	-.248	.309
	Hedges' correction	9.64235	.031	-.247	.308
	Glass's delta	9.99454	.030	-.249	.308
VE.stpd._0.8	Cohen's d	13.57222	-.020	-.299	.258
	Hedges' correction	13.62444	-.020	-.298	.257
	Glass's delta	13.83140	-.020	-.299	.259
VE.stpd._1	Cohen's d	21.52616	.035	-.243	.314
	Hedges' correction	21.60897	.035	-.242	.313
	Glass's delta	20.23427	.038	-.241	.316

a. The denominator used in estimating the effect sizes.
Cohen's d uses the pooled standard deviation.
Hedges' correction uses the pooled standard deviation, plus a correction factor.
Glass's delta uses the sample standard deviation of the control group.

Group Statistics

	Group	N	Mean	Std. Deviation	Std. Error Mean
RR_0.2	ME_CFS	178	18.5589	4.93953	.37023
	Control	169	20.1382	4.53976	.34921
RR_0.4	ME_CFS	178	19.4621	4.71496	.35340
	Control	169	21.4007	4.97424	.38263
RR_0.6	ME_CFS	178	22.0461	5.19020	.38902
	Control	169	24.6709	5.24375	.40337
RR_0.8	ME_CFS	178	25.5124	5.58673	.41874
	Control	169	29.4715	6.26557	.48197
RR_1	ME_CFS	178	35.3716	14.22821	1.06645
	Control	169	38.9105	8.79586	.67660

Independent Samples Effect Sizes

		Standardizer[a]	Point Estimate	95% Confidence Interval	
				Lower	Upper
RR_0.2	Cohen's d	4.74906	-.333	-.544	-.120
	Hedges' correction	4.75942	-.332	-.543	-.120
	Glass's delta	4.53976	-.348	-.561	-.134
RR_0.4	Cohen's d	4.84295	-.400	-.613	-.187
	Hedges' correction	4.85351	-.399	-.611	-.187
	Glass's delta	4.97424	-.390	-.604	-.175
RR_0.6	Cohen's d	5.21635	-.503	-.717	-.289
	Hedges' correction	5.22772	-.502	-.715	-.288
	Glass's delta	5.24375	-.501	-.717	-.283
RR_0.8	Cohen's d	5.92702	-.668	-.884	-.451
	Hedges' correction	5.93994	-.667	-.882	-.450
	Glass's delta	6.26557	-.632	-.852	-.410
RR_1	Cohen's d	11.89689	-.297	-.509	-.086
	Hedges' correction	11.92283	-.297	-.508	-.085
	Glass's delta	8.79586	-.402	-.617	-.187

a. The denominator used in estimating the effect sizes.
Cohen's d uses the pooled standard deviation.
Hedges' correction uses the pooled standard deviation, plus a correction factor.
Glass's delta uses the sample standard deviation of the control group.

Group Statistics

	Group	N	Mean	Std. Deviation	Std. Error Mean
RR_0.2	ME_CFS	99	17.9939	5.05541	.50809
	Control	99	20.0441	5.01187	.50371
RR_0.4	ME_CFS	99	18.9745	4.77093	.47950
	Control	99	21.2234	5.18235	.52085
RR_0.6	ME_CFS	99	21.4469	5.23890	.52653
	Control	99	24.3329	5.42374	.54511
RR_0.8	ME_CFS	99	24.6798	5.40232	.54295
	Control	99	28.8757	6.76475	.67988
RR_1	ME_CFS	99	33.6946	10.14428	1.01954
	Control	99	37.4464	9.17512	.92213

Independent Samples Effect Sizes

		Standardizer[a]	Point Estimate	95% Confidence Interval	
				Lower	Upper
RR_0.2	Cohen's d	5.03369	-.407	-.688	-.125
	Hedges' correction	5.05305	-.406	-.686	-.125
	Glass's delta	5.01187	-.409	-.692	-.124
RR_0.4	Cohen's d	4.98089	-.451	-.733	-.169
	Hedges' correction	5.00005	-.450	-.730	-.168
	Glass's delta	5.18235	-.434	-.718	-.148
RR_0.6	Cohen's d	5.33212	-.541	-.824	-.257
	Hedges' correction	5.35263	-.539	-.821	-.256
	Glass's delta	5.42374	-.532	-.819	-.242
RR_0.8	Cohen's d	6.12155	-.685	-.971	-.398
	Hedges' correction	6.14510	-.683	-.968	-.396
	Glass's delta	6.76475	-.620	-.911	-.327
RR_1	Cohen's d	9.67184	-.388	-.669	-.106
	Hedges' correction	9.70905	-.386	-.666	-.106
	Glass's delta	9.17512	-.409	-.692	-.124

a. The denominator used in estimating the effect sizes.
Cohen's d uses the pooled standard deviation.
Hedges' correction uses the pooled standard deviation, plus a correction factor.
Glass's delta uses the sample standard deviation of the control group.

Group Statistics

	Group	N	Mean	Std. Deviation	Std. Error Mean
HR_0.2	ME_CFS	178	97.1603	18.24059	1.36719
	Control	169	95.2028	16.98349	1.30642
HR_0.4	ME_CFS	178	109.1412	18.41148	1.38000
	Control	169	109.0935	15.36370	1.18182
HR_0.6	ME_CFS	178	123.1501	19.34885	1.45026
	Control	169	128.7629	22.73335	1.74872
HR_0.8	ME_CFS	178	139.4390	22.79013	1.70819
	Control	169	146.2622	22.97296	1.76715
HR_1	ME_CFS	178	158.8131	23.25826	1.74328
	Control	169	171.2411	25.33109	1.94855

Independent Samples Effect Sizes

		Standardizer[a]	Point Estimate	95% Confidence Interval	
				Lower	Upper
HR_0.2	Cohen's d	17.63963	.111	-.100	.322
	Hedges' correction	17.67809	.111	-.100	.321
	Glass's delta	16.98349	.115	-.096	.326
HR_0.4	Cohen's d	16.99575	.003	-.208	.213
	Hedges' correction	17.03281	.003	-.207	.213
	Glass's delta	15.36370	.003	-.207	.214
HR_0.6	Cohen's d	21.06499	-.266	-.478	-.055
	Hedges' correction	21.11092	-.266	-.477	-.055
	Glass's delta	22.73335	-.247	-.459	-.034
HR_0.8	Cohen's d	22.87934	-.298	-.510	-.086
	Hedges' correction	22.92923	-.298	-.509	-.086
	Glass's delta	22.97296	-.297	-.509	-.084
HR_1	Cohen's d	24.28975	-.512	-.725	-.297
	Hedges' correction	24.34271	-.511	-.724	-.297
	Glass's delta	25.33109	-.491	-.707	-.273

a. The denominator used in estimating the effect sizes.
Cohen's d uses the pooled standard deviation.
Hedges' correction uses the pooled standard deviation, plus a correction factor.
Glass's delta uses the sample standard deviation of the control group.

Group Statistics

	Group	N	Mean	Std. Deviation	Std. Error Mean
HR_0.2	ME_CFS	99	97.5491	15.38150	1.54590
	Control	99	95.7697	18.84526	1.89402
HR_0.4	ME_CFS	99	109.3384	15.98519	1.60657
	Control	99	106.8647	15.71391	1.57931
HR_0.6	ME_CFS	99	123.3685	18.56534	1.86589
	Control	99	125.2687	24.92203	2.50476
HR_0.8	ME_CFS	99	139.1990	20.35420	2.04567
	Control	99	139.8745	24.11430	2.42358
HR_1	ME_CFS	99	160.4229	21.93984	2.20504
	Control	99	165.3017	22.22405	2.23360

Independent Samples Effect Sizes

		Standardizer[a]	Point Estimate	95% Confidence Interval	
				Lower	Upper
HR_0.2	Cohen's d	17.20079	.103	-.175	.382
	Hedges' correction	17.26696	.103	-.175	.381
	Glass's delta	18.84526	.094	-.185	.373
HR_0.4	Cohen's d	15.85013	.156	-.123	.435
	Hedges' correction	15.91111	.155	-.123	.433
	Glass's delta	15.71391	.157	-.122	.436
HR_0.6	Cohen's d	21.97475	-.086	-.365	.192
	Hedges' correction	22.05929	-.086	-.364	.192
	Glass's delta	24.92203	-.076	-.355	.203
HR_0.8	Cohen's d	22.31359	-.030	-.309	.248
	Hedges' correction	22.39943	-.030	-.308	.247
	Glass's delta	24.11430	-.028	-.307	.251
HR_1	Cohen's d	22.08240	-.221	-.500	.059
	Hedges' correction	22.16735	-.220	-.498	.059
	Glass's delta	22.22405	-.220	-.499	.061

a. The denominator used in estimating the effect sizes.
Cohen's d uses the pooled standard deviation.
Hedges' correction uses the pooled standard deviation, plus a correction factor.
Glass's delta uses the sample standard deviation of the control group.

Group Statistics

	Group	N	Mean	Std. Deviation	Std. Error Mean
VE.VO2_0.2	ME_CFS	178	25.3833	5.46698	.40977
	Control	169	23.2084	3.77724	.29056
VE.VO2_0.4	ME_CFS	178	26.2482	5.75049	.43102
	Control	169	23.5947	3.52056	.27081
VE.VO2_0.6	ME_CFS	178	28.4070	6.27180	.47009
	Control	169	25.4908	4.09664	.31513
VE.VO2_0.8	ME_CFS	178	31.8684	7.52154	.56376
	Control	169	28.4145	4.84927	.37302
VE.VO2_1	ME_CFS	178	38.6419	9.95461	.74613
	Control	169	33.9573	6.19580	.47660

Independent Samples Effect Sizes

		Standardizer[a]	Point Estimate	95% Confidence Interval	
				Lower	Upper
VE.VO2_0.2	Cohen's d	4.72032	.461	.247	.674
	Hedges' correction	4.73062	.460	.247	.672
	Glass's delta	3.77724	.576	.356	.794
VE.VO2_0.4	Cohen's d	4.79593	.553	.338	.767
	Hedges' correction	4.80638	.552	.338	.766
	Glass's delta	3.52056	.754	.527	.978
VE.VO2_0.6	Cohen's d	5.32476	.548	.333	.762
	Hedges' correction	5.33637	.546	.332	.760
	Glass's delta	4.09664	.712	.487	.935
VE.VO2_0.8	Cohen's d	6.36205	.543	.328	.757
	Hedges' correction	6.37592	.542	.327	.755
	Glass's delta	4.84927	.712	.487	.935
VE.VO2_1	Cohen's d	8.33864	.562	.347	.776
	Hedges' correction	8.35682	.561	.346	.774
	Glass's delta	6.19580	.756	.530	.981

a. The denominator used in estimating the effect sizes.
Cohen's d uses the pooled standard deviation.
Hedges' correction uses the pooled standard deviation, plus a correction factor.
Glass's delta uses the sample standard deviation of the control group.

Group Statistics

	Group	N	Mean	Std. Deviation	Std. Error Mean
VE.VO2_0.2	ME_CFS	99	25.0282	5.62090	.56492
	Control	99	23.2259	3.89361	.39132
VE.VO2_0.4	ME_CFS	99	25.7741	5.50871	.55365
	Control	99	23.6218	4.03957	.40599
VE.VO2_0.6	ME_CFS	99	27.6846	5.78209	.58112
	Control	99	25.7872	4.63931	.46627
VE.VO2_0.8	ME_CFS	99	30.7601	6.67231	.67059
	Control	99	28.7494	5.35400	.53810
VE.VO2_1	ME_CFS	99	37.4150	9.12144	.91674
	Control	99	33.6350	6.71615	.67500

Independent Samples Effect Sizes

		Standardizer[a]	Point Estimate	95% Confidence Interval	
				Lower	Upper
VE.VO2_0.2	Cohen's d	4.83502	.373	.091	.653
	Hedges' correction	4.85362	.371	.091	.651
	Glass's delta	3.89361	.463	.176	.748
VE.VO2_0.4	Cohen's d	4.83032	.446	.163	.727
	Hedges' correction	4.84890	.444	.162	.724
	Glass's delta	4.03957	.533	.243	.820
VE.VO2_0.6	Cohen's d	5.24193	.362	.081	.642
	Hedges' correction	5.26210	.361	.080	.640
	Glass's delta	4.63931	.409	.124	.692
VE.VO2_0.8	Cohen's d	6.04918	.332	.051	.612
	Hedges' correction	6.07245	.331	.051	.610
	Glass's delta	5.35400	.376	.091	.658
VE.VO2_1	Cohen's d	8.00960	.472	.189	.754
	Hedges' correction	8.04041	.470	.188	.751
	Glass's delta	6.71615	.563	.272	.851

a. The denominator used in estimating the effect sizes.
Cohen's d uses the pooled standard deviation.
Hedges' correction uses the pooled standard deviation, plus a correction factor.
Glass's delta uses the sample standard deviation of the control group.

Group Statistics

	Group	N	Mean	Std. Deviation	Std. Error Mean
VE.VCO2_0.2	ME_CFS	178	31.3155	6.57783	.49303
	Control	169	29.4115	3.94041	.30311
VE.VCO2_0.4	ME_CFS	178	29.6761	6.52082	.48876
	Control	169	27.0624	3.05346	.23488
VE.VCO2_0.6	ME_CFS	178	28.8552	6.08999	.45646
	Control	169	26.2532	3.14070	.24159
VE.VCO2_0.8	ME_CFS	178	29.4958	6.42567	.48162
	Control	169	26.8188	3.63640	.27972
VE.VCO2_1	ME_CFS	178	32.8617	7.85433	.58871
	Control	169	29.5744	4.72186	.36322

Independent Samples Effect Sizes

		Standardizer[a]	Point Estimate	95% Confidence Interval	
				Lower	Upper
VE.VCO2_0.2	Cohen's d	5.45519	.349	.137	.561
	Hedges' correction	5.46709	.348	.136	.560
	Glass's delta	3.94041	.483	.266	.699
VE.VCO2_0.4	Cohen's d	5.13375	.509	.295	.723
	Hedges' correction	5.14494	.508	.294	.721
	Glass's delta	3.05346	.856	.625	1.084
VE.VCO2_0.6	Cohen's d	4.88171	.533	.318	.747
	Hedges' correction	4.89235	.532	.318	.745
	Glass's delta	3.14070	.828	.599	1.056
VE.VCO2_0.8	Cohen's d	5.25570	.509	.295	.723
	Hedges' correction	5.26716	.508	.294	.721
	Glass's delta	3.63640	.736	.510	.960
VE.VCO2_1	Cohen's d	6.51974	.504	.290	.718
	Hedges' correction	6.53396	.503	.289	.716
	Glass's delta	4.72186	.696	.472	.919

a. The denominator used in estimating the effect sizes.
Cohen's d uses the pooled standard deviation.
Hedges' correction uses the pooled standard deviation, plus a correction factor.
Glass's delta uses the sample standard deviation of the control group.

Group Statistics

	Group	N	Mean	Std. Deviation	Std. Error Mean
VE.VCO2_0.2	ME_CFS	99	30.9440	6.94541	.69804
	Control	99	29.1433	3.29130	.33079
VE.VCO2_0.4	ME_CFS	99	29.1037	6.23849	.62699
	Control	99	27.1471	3.20527	.32214
VE.VCO2_0.6	ME_CFS	99	28.2855	5.72967	.57585
	Control	99	26.3715	3.29580	.33124
VE.VCO2_0.8	ME_CFS	99	28.7351	5.87708	.59067
	Control	99	26.8424	3.68652	.37051
VE.VCO2_1	ME_CFS	99	32.0801	7.37232	.74095
	Control	99	29.0975	4.76021	.47842

Independent Samples Effect Sizes

		Standardizer[a]	Point Estimate	95% Confidence Interval	
				Lower	Upper
VE.VCO2_0.2	Cohen's d	5.43468	.331	.050	.611
	Hedges' correction	5.45558	.330	.050	.609
	Glass's delta	3.29130	.547	.257	.835
VE.VCO2_0.4	Cohen's d	4.95946	.395	.113	.675
	Hedges' correction	4.97854	.393	.112	.673
	Glass's delta	3.20527	.610	.318	.900
VE.VCO2_0.6	Cohen's d	4.67394	.410	.127	.691
	Hedges' correction	4.69192	.408	.127	.688
	Glass's delta	3.29580	.581	.289	.870
VE.VCO2_0.8	Cohen's d	4.90563	.386	.104	.667
	Hedges' correction	4.92451	.384	.104	.664
	Glass's delta	3.68652	.513	.225	.800
VE.VCO2_1	Cohen's d	6.20527	.481	.197	.763
	Hedges' correction	6.22914	.479	.197	.760
	Glass's delta	4.76021	.627	.333	.917

a. The denominator used in estimating the effect sizes.
Cohen's d uses the pooled standard deviation.
Hedges' correction uses the pooled standard deviation, plus a correction factor.
Glass's delta uses the sample standard deviation of the control group.

Group Statistics

	Group	N	Mean	Std. Deviation	Std. Error Mean
TV.stpd._0.2	ME_CFS	178	.8899	.31482	.02360
	Control	169	.8065	.28623	.02202
TV.stpd._0.4	ME_CFS	178	1.1264	.37767	.02831
	Control	169	1.0820	.40718	.03132
TV.stpd._0.6	ME_CFS	178	1.3542	.45963	.03445
	Control	169	1.3294	.47976	.03690
TV.stpd._0.8	ME_CFS	178	1.5806	.53060	.03977
	Control	169	1.5597	.54321	.04179
TV.stpd._1	ME_CFS	178	1.6524	.55485	.04159
	Control	169	1.6506	.54791	.04215

Independent Samples Effect Sizes

		Standardizer[a]	Point Estimate	95% Confidence Interval	
				Lower	Upper
TV.stpd._0.2	Cohen's d	.30124	.277	.065	.488
	Hedges' correction	.30189	.276	.065	.487
	Glass's delta	.28623	.292	.078	.504
TV.stpd._0.4	Cohen's d	.39232	.113	-.098	.324
	Hedges' correction	.39317	.113	-.097	.323
	Glass's delta	.40718	.109	-.102	.320
TV.stpd._0.6	Cohen's d	.46954	.053	-.158	.263
	Hedges' correction	.47056	.053	-.158	.263
	Glass's delta	.47976	.052	-.159	.262
TV.stpd._0.8	Cohen's d	.53677	.039	-.172	.249
	Hedges' correction	.53795	.039	-.171	.249
	Glass's delta	.54321	.039	-.172	.249
TV.stpd._1	Cohen's d	.55148	.003	-.207	.214
	Hedges' correction	.55268	.003	-.207	.213
	Glass's delta	.54791	.003	-.207	.214

a. The denominator used in estimating the effect sizes.
Cohen's d uses the pooled standard deviation.
Hedges' correction uses the pooled standard deviation, plus a correction factor.
Glass's delta uses the sample standard deviation of the control group.

Group Statistics

	Group	N	Mean	Std. Deviation	Std. Error Mean
TV.stpd._0.2	ME_CFS	99	.9390	.33315	.03348
	Control	99	.7421	.26519	.02665
TV.stpd._0.4	ME_CFS	99	1.1994	.40159	.04036
	Control	99	.9929	.37887	.03808
TV.stpd._0.6	ME_CFS	99	1.4481	.48338	.04858
	Control	99	1.2319	.44128	.04435
TV.stpd._0.8	ME_CFS	99	1.7013	.56514	.05680
	Control	99	1.4620	.52955	.05322
TV.stpd._1	ME_CFS	99	1.7616	.58632	.05893
	Control	99	1.5298	.53682	.05395

Independent Samples Effect Sizes

		Standardizer[a]	Point Estimate	95% Confidence Interval	
				Lower	Upper
TV.stpd._0.2	Cohen's d	.30110	.654	.367	.939
	Hedges' correction	.30225	.651	.366	.935
	Glass's delta	.26519	.742	.443	1.038
TV.stpd._0.4	Cohen's d	.39039	.529	.245	.812
	Hedges' correction	.39189	.527	.244	.809
	Glass's delta	.37887	.545	.255	.833
TV.stpd._0.6	Cohen's d	.46281	.467	.184	.749
	Hedges' correction	.46459	.465	.184	.746
	Glass's delta	.44128	.490	.202	.776
TV.stpd._0.8	Cohen's d	.54763	.437	.155	.718
	Hedges' correction	.54974	.435	.154	.716
	Glass's delta	.52955	.452	.165	.737
TV.stpd._1	Cohen's d	.56211	.412	.130	.693
	Hedges' correction	.56428	.411	.130	.691
	Glass's delta	.53682	.432	.146	.716

a. The denominator used in estimating the effect sizes.
Cohen's d uses the pooled standard deviation.
Hedges' correction uses the pooled standard deviation, plus a correction factor.
Glass's delta uses the sample standard deviation of the control group.

Group Statistics

	Group	N	Mean	Std. Deviation	Std. Error Mean
RPE_0.2	ME_CFS	147	10.1020	2.49585	.20585
	Control	149	9.3557	2.07323	.16985
RPE_0.4	ME_CFS	178	11.5090	2.22594	.16684
	Control	169	10.9609	2.29170	.17628
RPE_0.6	ME_CFS	178	13.7052	2.30149	.17250
	Control	169	13.1318	2.39965	.18459
RPE_0.8	ME_CFS	178	16.3874	2.03194	.15230
	Control	169	15.5794	2.34915	.18070
RPE_1	ME_CFS	178	19.1362	1.04897	.07862
	Control	169	18.3373	1.86170	.14321

Independent Samples Effect Sizes

		Standardizer[a]	Point Estimate	95% Confidence Interval	
				Lower	Upper
RPE_0.2	Cohen's d	2.29286	.326	.096	.555
	Hedges' correction	2.29873	.325	.096	.553
	Glass's delta	2.07323	.360	.128	.591
RPE_0.4	Cohen's d	2.25820	.243	.031	.454
	Hedges' correction	2.26313	.242	.031	.453
	Glass's delta	2.29170	.239	.027	.451
RPE_0.6	Cohen's d	2.34980	.244	.033	.455
	Hedges' correction	2.35493	.243	.032	.454
	Glass's delta	2.39965	.239	.027	.451
RPE_0.8	Cohen's d	2.19214	.369	.156	.581
	Hedges' correction	2.19692	.368	.156	.579
	Glass's delta	2.34915	.344	.130	.557
RPE_1	Cohen's d	1.50076	.532	.318	.746
	Hedges' correction	1.50403	.531	.317	.745
	Glass's delta	1.86170	.429	.213	.644

a. The denominator used in estimating the effect sizes.
Cohen's d uses the pooled standard deviation.
Hedges' correction uses the pooled standard deviation, plus a correction factor.
Glass's delta uses the sample standard deviation of the control group.

Group Statistics

	Group	N	Mean	Std. Deviation	Std. Error Mean
RPE_0.2	ME_CFS	83	10.4337	2.37463	.26065
	Control	84	9.1429	2.12355	.23170
RPE_0.4	ME_CFS	99	11.8071	2.33232	.23441
	Control	99	10.7745	2.45130	.24637
RPE_0.6	ME_CFS	99	14.0026	2.39261	.24047
	Control	99	12.9547	2.52003	.25327
RPE_0.8	ME_CFS	99	16.5704	2.07522	.20857
	Control	99	15.4338	2.34361	.23554
RPE_1	ME_CFS	99	19.1389	1.02034	.10255
	Control	99	18.3712	1.65422	.16626

Independent Samples Effect Sizes

		Standardizer[a]	Point Estimate	95% Confidence Interval	
				Lower	Upper
RPE_0.2	Cohen's d	2.25183	.573	.263	.882
	Hedges' correction	2.26213	.571	.262	.878
	Glass's delta	2.12355	.608	.289	.923
RPE_0.4	Cohen's d	2.39255	.432	.149	.713
	Hedges' correction	2.40176	.430	.149	.710
	Glass's delta	2.45130	.421	.135	.705
RPE_0.6	Cohen's d	2.45715	.426	.144	.708
	Hedges' correction	2.46660	.425	.144	.705
	Glass's delta	2.52003	.416	.130	.699
RPE_0.8	Cohen's d	2.21349	.513	.230	.796
	Hedges' correction	2.22200	.512	.229	.793
	Glass's delta	2.34361	.485	.197	.771
RPE_1	Cohen's d	1.37432	.559	.274	.842
	Hedges' correction	1.37961	.556	.273	.839
	Glass's delta	1.65422	.464	.177	.749

a. The denominator used in estimating the effect sizes.
Cohen's d uses the pooled standard deviation.
Hedges' correction uses the pooled standard deviation, plus a correction factor.
Glass's delta uses the sample standard deviation of the control group.

Group Statistics

	Group	N	Mean	Std. Deviation	Std. Error Mean
VO2.Watts_0.2	ME_CFS	178	.0194	.00869	.00065
	Control	169	.0173	.00554	.00043
VO2.Watts_0.4	ME_CFS	176	.0150	.00389	.00029
	Control	167	.0141	.00319	.00025
VO2.Watts_0.6	ME_CFS	178	.0137	.00289	.00022
	Control	167	.0132	.00233	.00018
VO2.Watts_0.8	ME_CFS	175	.0133	.00264	.00020
	Control	168	.0131	.00204	.00016
VO2.Watts_1	ME_CFS	178	.0130	.00256	.00019
	Control	169	.0128	.00190	.00015

Independent Samples Effect Sizes

		Standardizer[a]	Point Estimate	95% Confidence Interval	
				Lower	Upper
VO2.Watts_0.2	Cohen's d	.00733	.296	.084	.507
	Hedges' correction	.00735	.295	.084	.506
	Glass's delta	.00554	.391	.176	.605
VO2.Watts_0.4	Cohen's d	.00357	.246	.034	.459
	Hedges' correction	.00358	.246	.034	.458
	Glass's delta	.00319	.276	.061	.489
VO2.Watts_0.6	Cohen's d	.00263	.193	-.018	.405
	Hedges' correction	.00264	.193	-.018	.404
	Glass's delta	.00233	.219	.006	.431
VO2.Watts_0.8	Cohen's d	.00237	.060	-.152	.272
	Hedges' correction	.00237	.060	-.151	.271
	Glass's delta	.00204	.070	-.142	.282
VO2.Watts_1	Cohen's d	.00226	.056	-.154	.267
	Hedges' correction	.00227	.056	-.154	.266
	Glass's delta	.00190	.067	-.144	.278

a. The denominator used in estimating the effect sizes.
Cohen's d uses the pooled standard deviation.
Hedges' correction uses the pooled standard deviation, plus a correction factor.
Glass's delta uses the sample standard deviation of the control group.

Group Statistics

	Group	N	Mean	Std. Deviation	Std. Error Mean
VO2.Watts_0.2	ME_CFS	99	.0192	.00741	.00074
	Control	99	.0177	.00651	.00065
VO2.Watts_0.4	ME_CFS	99	.0151	.00346	.00035
	Control	98	.0141	.00381	.00038
VO2.Watts_0.6	ME_CFS	99	.0139	.00271	.00027
	Control	98	.0131	.00269	.00027
VO2.Watts_0.8	ME_CFS	97	.0135	.00266	.00027
	Control	98	.0130	.00232	.00023
VO2.Watts_1	ME_CFS	99	.0132	.00269	.00027
	Control	99	.0126	.00209	.00021

Independent Samples Effect Sizes

		Standardizer[a]	Point Estimate	95% Confidence Interval	
				Lower	Upper
VO2.Watts_0.2	Cohen's d	.00698	.211	-.068	.490
	Hedges' correction	.00700	.210	-.068	.488
	Glass's delta	.00651	.226	-.055	.506
VO2.Watts_0.4	Cohen's d	.00364	.290	.009	.570
	Hedges' correction	.00365	.289	.009	.568
	Glass's delta	.00381	.277	-.006	.558
VO2.Watts_0.6	Cohen's d	.00270	.309	.028	.590
	Hedges' correction	.00271	.308	.028	.588
	Glass's delta	.00269	.310	.027	.592
VO2.Watts_0.8	Cohen's d	.00250	.190	-.091	.471
	Hedges' correction	.00250	.190	-.091	.470
	Glass's delta	.00232	.204	-.078	.486
VO2.Watts_1	Cohen's d	.00241	.240	-.040	.519
	Hedges' correction	.00242	.239	-.040	.517
	Glass's delta	.00209	.276	-.006	.557

a. The denominator used in estimating the effect sizes.
Cohen's d uses the pooled standard deviation.
Hedges' correction uses the pooled standard deviation, plus a correction factor.
Glass's delta uses the sample standard deviation of the control group.

Group Statistics

	Group	N	Mean	Std. Deviation	Std. Error Mean
VO2.HR_0.2	ME_CFS	178	.0080	.00286	.00021
	Control	169	.0083	.00341	.00026
VO2.HR_0.4	ME_CFS	176	.0093	.00341	.00026
	Control	165	.0099	.00378	.00029
VO2.HR_0.6	ME_CFS	178	.0105	.00394	.00030
	Control	166	.0113	.00420	.00033
VO2.HR_0.8	ME_CFS	175	.0113	.00408	.00031
	Control	168	.0125	.00457	.00035
VO2.HR_1	ME_CFS	176	.0114	.00413	.00031
	Control	168	.0125	.00468	.00036

Independent Samples Effect Sizes

		Standardizer[a]	Point Estimate	95% Confidence Interval Lower	95% Confidence Interval Upper
VO2.HR_0.2	Cohen's d	.00314	-.116	-.327	.094
	Hedges' correction	.00315	-.116	-.326	.094
	Glass's delta	.00341	-.107	-.318	.104
VO2.HR_0.4	Cohen's d	.00360	-.166	-.379	.046
	Hedges' correction	.00361	-.166	-.378	.046
	Glass's delta	.00378	-.158	-.371	.055
VO2.HR_0.6	Cohen's d	.00406	-.198	-.410	.014
	Hedges' correction	.00407	-.198	-.409	.014
	Glass's delta	.00420	-.192	-.404	.021
VO2.HR_0.8	Cohen's d	.00433	-.268	-.480	-.055
	Hedges' correction	.00434	-.267	-.479	-.055
	Glass's delta	.00457	-.254	-.467	-.040
VO2.HR_1	Cohen's d	.00441	-.248	-.460	-.036
	Hedges' correction	.00442	-.247	-.459	-.035
	Glass's delta	.00468	-.234	-.446	-.020

a. The denominator used in estimating the effect sizes.
Cohen's d uses the pooled standard deviation.
Hedges' correction uses the pooled standard deviation, plus a correction factor.
Glass's delta uses the sample standard deviation of the control group.

Group Statistics

	Group	N	Mean	Std. Deviation	Std. Error Mean
VO2.HR_0.2	ME_CFS	99	.0081	.00281	.00028
	Control	99	.0075	.00365	.00037
VO2.HR_0.4	ME_CFS	99	.0097	.00349	.00035
	Control	98	.0091	.00398	.00040
VO2.HR_0.6	ME_CFS	99	.0110	.00412	.00041
	Control	97	.0104	.00431	.00044
VO2.HR_0.8	ME_CFS	97	.0119	.00421	.00043
	Control	98	.0116	.00481	.00049
VO2.HR_1	ME_CFS	98	.0120	.00420	.00042
	Control	98	.0114	.00439	.00044

Independent Samples Effect Sizes

		Standardizer[a]	Point Estimate	95% Confidence Interval	
				Lower	Upper
VO2.HR_0.2	Cohen's d	.00326	.174	-.106	.453
	Hedges' correction	.00327	.173	-.105	.451
	Glass's delta	.00365	.155	-.125	.434
VO2.HR_0.4	Cohen's d	.00374	.153	-.127	.433
	Hedges' correction	.00375	.153	-.126	.431
	Glass's delta	.00398	.144	-.136	.424
VO2.HR_0.6	Cohen's d	.00421	.145	-.135	.426
	Hedges' correction	.00423	.145	-.135	.424
	Glass's delta	.00431	.142	-.139	.422
VO2.HR_0.8	Cohen's d	.00452	.061	-.220	.341
	Hedges' correction	.00454	.060	-.219	.340
	Glass's delta	.00481	.057	-.224	.338
VO2.HR_1	Cohen's d	.00430	.150	-.131	.430
	Hedges' correction	.00431	.149	-.130	.428
	Glass's delta	.00439	.146	-.135	.427

a. The denominator used in estimating the effect sizes.
Cohen's d uses the pooled standard deviation.
Hedges' correction uses the pooled standard deviation, plus a correction factor.
Glass's delta uses the sample standard deviation of the control group.

Group Statistics

	Group	N	Mean	Std. Deviation	Std. Error Mean
CI_0.2	ME_CFS	178	.9922	.28717	.02152
	Control	169	1.0404	.21812	.01678
CI_0.4	ME_CFS	176	.9425	.12777	.00963
	Control	165	.9815	.13637	.01062
CI_0.6	ME_CFS	178	.9174	.11797	.00884
	Control	166	.9641	.16619	.01290
CI_0.8	ME_CFS	175	.9084	.12836	.00970
	Control	168	.9307	.12266	.00946
CI_1	ME_CFS	176	.9302	.11938	.00900
	Control	168	.9643	.12115	.00935

Independent Samples Effect Sizes

		Standardizer[a]	Point Estimate	95% Confidence Interval	
				Lower	Upper
CI_0.2	Cohen's d	.25588	-.188	-.399	.023
	Hedges' correction	.25644	-.188	-.398	.023
	Glass's delta	.21812	-.221	-.433	-.009
CI_0.4	Cohen's d	.13200	-.295	-.509	-.082
	Hedges' correction	.13229	-.295	-.508	-.081
	Glass's delta	.13637	-.286	-.500	-.071
CI_0.6	Cohen's d	.14327	-.326	-.538	-.113
	Hedges' correction	.14359	-.325	-.537	-.112
	Glass's delta	.16619	-.281	-.494	-.067
CI_0.8	Cohen's d	.12560	-.178	-.390	.035
	Hedges' correction	.12588	-.177	-.389	.035
	Glass's delta	.12266	-.182	-.394	.031
CI_1	Cohen's d	.12025	-.283	-.496	-.071
	Hedges' correction	.12052	-.283	-.494	-.070
	Glass's delta	.12115	-.281	-.494	-.067

a. The denominator used in estimating the effect sizes.
Cohen's d uses the pooled standard deviation.
Hedges' correction uses the pooled standard deviation, plus a correction factor.
Glass's delta uses the sample standard deviation of the control group.

Group Statistics

	Group	N	Mean	Std. Deviation	Std. Error Mean
CI_0.2	ME_CFS	99	1.0037	.33532	.03370
	Control	99	1.0742	.25342	.02547
CI_0.4	ME_CFS	99	.9371	.09812	.00986
	Control	98	.9895	.15480	.01564
CI_0.6	ME_CFS	99	.9097	.10659	.01071
	Control	97	.9618	.19846	.02015
CI_0.8	ME_CFS	97	.8962	.10258	.01042
	Control	98	.9137	.14035	.01418
CI_1	ME_CFS	98	.9296	.10752	.01086
	Control	98	.9550	.11308	.01142

Independent Samples Effect Sizes

		Standardizer[a]	Point Estimate	95% Confidence Interval	
				Lower	Upper
CI_0.2	Cohen's d	.29720	-.237	-.516	.043
	Hedges' correction	.29834	-.236	-.514	.042
	Glass's delta	.25342	-.278	-.559	.004
CI_0.4	Cohen's d	.12946	-.405	-.687	-.122
	Hedges' correction	.12996	-.404	-.684	-.122
	Glass's delta	.15480	-.339	-.621	-.055
CI_0.6	Cohen's d	.15884	-.328	-.609	-.046
	Hedges' correction	.15945	-.327	-.607	-.045
	Glass's delta	.19846	-.262	-.544	.021
CI_0.8	Cohen's d	.12302	-.142	-.423	.139
	Hedges' correction	.12350	-.141	-.421	.139
	Glass's delta	.14035	-.124	-.405	.157
CI_1	Cohen's d	.11033	-.231	-.511	.051
	Hedges' correction	.11076	-.230	-.509	.050
	Glass's delta	.11308	-.225	-.506	.057

a. The denominator used in estimating the effect sizes.
Cohen's d uses the pooled standard deviation.
Hedges' correction uses the pooled standard deviation, plus a correction factor.
Glass's delta uses the sample standard deviation of the control group.

Group Statistics

	Group	N	Mean	Std. Deviation	Std. Error Mean
Work.VO2.Relation_0.2	ME_CFS	178	.0062	.00435	.00033
	Control	167	.0072	.00394	.00031
Work.VO2.Relation_0.4	ME_CFS	176	.0070	.00273	.00021
	Control	165	.0080	.00232	.00018
Work.VO2.Relation_0.6	ME_CFS	178	.0079	.00223	.00017
	Control	166	.0088	.00177	.00014
Work.VO2.Relation_0.8	ME_CFS	175	.0086	.00229	.00017
	Control	168	.0096	.00171	.00013
Work.VO2.Relation_1	ME_CFS	178	.0090	.00238	.00018
	Control	169	.0100	.00169	.00013

Independent Samples Effect Sizes

				95% Confidence Interval	
		Standardizer[a]	Point Estimate	Lower	Upper
Work.VO2.Relation_0.2	Cohen's d	.00416	-.223	-.435	-.011
	Hedges' correction	.00417	-.223	-.434	-.011
	Glass's delta	.00394	-.236	-.448	-.023
Work.VO2.Relation_0.4	Cohen's d	.00254	-.391	-.605	-.176
	Hedges' correction	.00255	-.390	-.604	-.176
	Glass's delta	.00232	-.428	-.645	-.210
Work.VO2.Relation_0.6	Cohen's d	.00202	-.436	-.650	-.222
	Hedges' correction	.00202	-.435	-.648	-.221
	Glass's delta	.00177	-.497	-.715	-.278
Work.VO2.Relation_0.8	Cohen's d	.00202	-.513	-.727	-.297
	Hedges' correction	.00203	-.511	-.726	-.296
	Glass's delta	.00171	-.607	-.827	-.384
Work.VO2.Relation_1	Cohen's d	.00207	-.492	-.706	-.278
	Hedges' correction	.00208	-.491	-.704	-.278
	Glass's delta	.00169	-.604	-.824	-.383

a. The denominator used in estimating the effect sizes.
Cohen's d uses the pooled standard deviation.
Hedges' correction uses the pooled standard deviation, plus a correction factor.
Glass's delta uses the sample standard deviation of the control group.

Group Statistics

	Group	N	Mean	Std. Deviation	Std. Error Mean
Work.VO2.Relation_0.2	ME_CFS	99	.0063	.00414	.00042
	Control	97	.0072	.00479	.00049
Work.VO2.Relation_0.4	ME_CFS	99	.0073	.00238	.00024
	Control	98	.0078	.00272	.00028
Work.VO2.Relation_0.6	ME_CFS	99	.0082	.00212	.00021
	Control	97	.0084	.00188	.00019
Work.VO2.Relation_0.8	ME_CFS	97	.0089	.00229	.00023
	Control	98	.0094	.00183	.00019
Work.VO2.Relation_1	ME_CFS	99	.0093	.00238	.00024
	Control	99	.0097	.00173	.00017

Independent Samples Effect Sizes

		Standardizer[a]	Point Estimate	95% Confidence Interval	
				Lower	Upper
Work.VO2.Relation_0.2	Cohen's d	.00447	-.209	-.489	.072
	Hedges' correction	.00449	-.208	-.488	.072
	Glass's delta	.00479	-.195	-.476	.087
Work.VO2.Relation_0.4	Cohen's d	.00256	-.203	-.483	.077
	Hedges' correction	.00257	-.203	-.481	.077
	Glass's delta	.00272	-.191	-.471	.090
Work.VO2.Relation_0.6	Cohen's d	.00201	-.142	-.422	.139
	Hedges' correction	.00201	-.141	-.420	.138
	Glass's delta	.00188	-.152	-.432	.130
Work.VO2.Relation_0.8	Cohen's d	.00207	-.223	-.504	.059
	Hedges' correction	.00208	-.222	-.502	.059
	Glass's delta	.00183	-.252	-.534	.032
Work.VO2.Relation_1	Cohen's d	.00208	-.202	-.481	.078
	Hedges' correction	.00209	-.201	-.479	.077
	Glass's delta	.00173	-.243	-.523	.039

a. The denominator used in estimating the effect sizes.
Cohen's d uses the pooled standard deviation.
Hedges' correction uses the pooled standard deviation, plus a correction factor.
Glass's delta uses the sample standard deviation of the control group.
