# Supplementary material for: Cardiopulmonary, metabolic, and perceptual responses during exercise in Myalgic Encephalomyelitis/Chronic Fatigue Syndrome (ME/CFS): A Multi-site Clinical Assessment of ME/CFS (MCAM) sub-study
Source: PLoS One. 2022 Mar 15;17(3):e0265315. doi: 10.1371/journal.pone.0265315 (PMC8923458; doi:10.1371/journal.pone.0265315)

**Supplemental Figure Legend:** Lactate responses at rest, minute-2 of exercise, peak exercise and at 3, 6 and 9-minutes post-exercise for participants with ME/CFS and otherwise healthy controls. There were no significant differences in lactate responses between groups for either the whole sample or when.


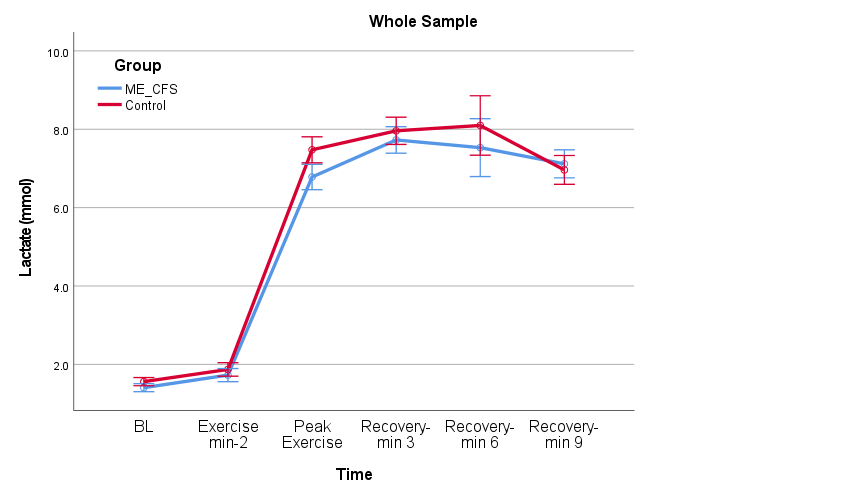


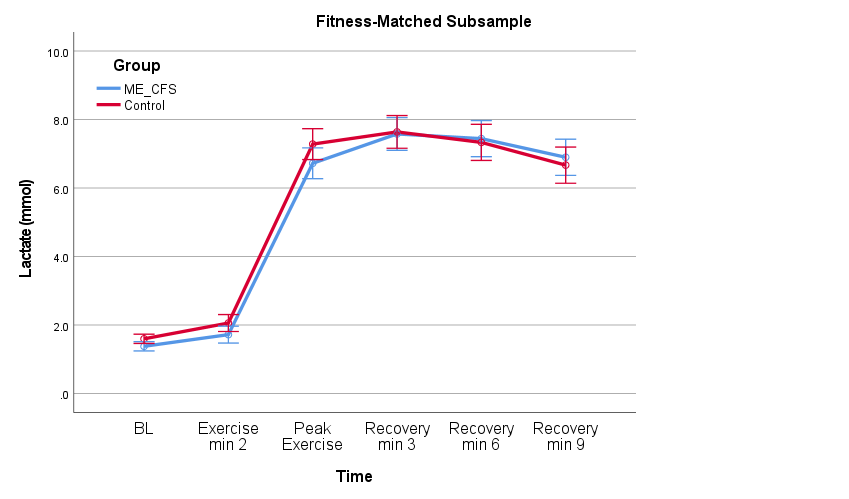

Supplement: S1 Fig — (DOCX) [file pone.0265315.s004.docx]
