# Supplementary material for: Cardiopulmonary, metabolic, and perceptual responses during exercise in Myalgic Encephalomyelitis/Chronic Fatigue Syndrome (ME/CFS): A Multi-site Clinical Assessment of ME/CFS (MCAM) sub-study
Source: PLoS One. 2022 Mar 15;17(3):e0265315. doi: 10.1371/journal.pone.0265315 (PMC8923458; doi:10.1371/journal.pone.0265315)
Supplement: S1 Table — (DOCX) [file pone.0265315.s005.docx]

Supplementary Table 1. – Characteristic of participants with ME/CFS – Overall Functioning and Symptom Status

|  | **Mean** | **Std Dev** | **Std Error** | **Minimum** | **Median** | **Upper Quartile** | **Maximum** |
| --- | --- | --- | --- | --- | --- | --- | --- |
| **MFI-20 Subscales** |  |  |  |  |  |  |  |
| General Fatigue | 17.42 | 2.97 | 0.23 | 8 | 18 | 20 | 20 |
| Physical Fatigue | 16.8 | 3.38 | 0.26 | 6 | 18 | 20 | 20 |
| Reduced Activity | 15.88 | 3.86 | 0.3 | 6 | 17 | 20 | 20 |
| Reduced Motivation | 11.06 | 3.87 | 0.3 | 4 | 10 | 14 | 20 |
| Mental Fatigue | 14.25 | 4.1 | 0.32 | 4 | 14 | 17 | 20 |
| **SF-36 Subscales (0-100)** |  |  |  |  |  |  |  |
| Physical Functioning | 45.98 | 24.01 | 1.87 | 0 | 40 | 65 | 100 |
| Physical | 23.53 | 34.8 | 2.67 | 0 | 0 | 50 | 100 |
| Bodily Pain | 44.7 | 26.43 | 2.03 | 0 | 41 | 62 | 100 |
| Social Functioning | 31.59 | 26.81 | 2.07 | 0 | 25 | 50 | 100 |
| Mental Health | 66.54 | 21.09 | 1.63 | 4 | 72 | 84 | 100 |
| Role Emotional | 77.45 | 39.66 | 3.04 | 0 | 100 | 100 | 100 |
| Vitality | 21.07 | 19.03 | 1.47 | 0 | 15 | 30 | 85 |
| General Health | 28.75 | 18.03 | 1.39 | 0 | 25 | 37 | 87 |
| **CDC-SI** |  |  |  |  |  |  |  |
| No. of CFS Symptoms (0-80) | 5.58 | 1.95 | 0.17 | 0 | 6 | 7 | 8 |
| CFS Symptom Score (0-128) | 47.64 | 22.57 | 1.9 | 0 | 48 | 63 | 98.5 |
| **PROMIS T-Scores** |  |  |  |  |  |  |  |
| Fatigue | 66.11 | 7.53 | 0.58 | 43.9 | 66.3 | 71.1 | 83.2 |
| Sleep Disturbance | 58.43 | 8.05 | 0.62 | 39.8 | 59.4 | 64.9 | 76.5 |
| Sleep Related Impairment | 61.25 | 7.93 | 0.61 | 41.4 | 62.3 | 67.3 | 80.0 |
| Pain Intensity | 59.53 | 9.96 | 0.76 | 41.0 | 60.9 | 66.4 | 78.3 |
| Pain Behavior | 55.93 | 8.39 | 0.65 | 36.7 | 58.6 | 61.2 | 65.8 |
