# Supplementary material for: Cardiopulmonary, metabolic, and perceptual responses during exercise in Myalgic Encephalomyelitis/Chronic Fatigue Syndrome (ME/CFS): A Multi-site Clinical Assessment of ME/CFS (MCAM) sub-study
Source: PLoS One. 2022 Mar 15;17(3):e0265315. doi: 10.1371/journal.pone.0265315 (PMC8923458; doi:10.1371/journal.pone.0265315)
Supplement: Attachment — Submitted filename: Response to Reviewers.docx [file pone.0265315.s006.docx]

**We would like to thank the reviewers for their thoughtful and thorough review of our work. Please find our point-by-point response below. Where relevant, we have included the changes within our response to aid the reviewer and we have indicated where in the track changes document each change has been made.**

**Annotated Reviews for MCAM paper:**

**Reviewer #1: This study examined cardiorespiratory responses to exercise in a group with Myalgic**

Encephalomyelitis/Chronic Fatigue Syndrome (ME/CFS) compared to a control group. Although I value the amount of work put into this manuscript, I have some major concerns as described in detail below. As a general comment, I believe that the methodology used in this study does not allow to gain the mechanistic insights that are proposed in the manuscript. This leads to an overly speculative discussion that is not supported by the data the authors presented.

*We have addressed the reviewer’s concern about being “overly speculative” throughout the discussion (See our responses below). Specifically, we have focused more on what types of future research are needed to test the mechanisms that might underlie the exercise response differences that we observed, and the need for more research to determine the clinical relevance of our data. For example, we have removed the phrase “suggestive of preserved oxygen delivery but poor oxygen utilization” from both the abstract (Page 2, lines 57 & 58) and the discussion (Page 15, line 416), have removed the paragraph discussing metaboreflex and redistribution of peripheral blood flow (Page 17, lines 495-510), and removed language gas exchange and other potential explanations for the increased exertion ratings (Page 17, lines 519 & 520).*

*We respectfully disagree that cardiopulmonary exercise testing (CPET) does not afford mechanistic insight. The Reviewer’s opinion is counter to expert consensus from the American Thoracic Society and American College of Chest Physicians (ATS/ACCP). As described by ATS/ACCP, there are numerous specific indications for CPET (c.f., Table 1 in* [*ATS/ACCP*](https://www.atsjournals.org/doi/abs/10.1164/rccm.167.2.211)*). Of relevance for ME/CFS is the evaluation of undiagnosed exercise intolerance, particularly when “symptoms are disproportionate to resting pulmonary and cardiac tests” and that “[CPET affords] Determination of exercise-limiting factors and pathophysiologic mechanisms.” Though indicated, CPET interpretation is challenging even for experienced clinicians given the vast amount of data. There have been numerous attempts to fit CPET data into overly simplistic algorithms using single value thresholds for select parameters. Such approaches are counterproductive as the utility of CPET lies in the integrated response to exercise. In fact, there is consensus amongst current experts recommending that the researcher or clinician “…should recognize patterns of dysfunction based on clusters of variables rather than relying on rigid interpretative algorithms”* ***(***[***Neder et al. 2021***](https://www.frontiersin.org/articles/10.3389/fphys.2021.552000/full)***).*** *Patterns of dysfunction were first popularized by Dr. Wasserman’s 9-panel plots (*[***Wasserman et al. 1997***](https://pubmed.ncbi.nlm.nih.gov/9377922/)*) and most recently have taken the form of condition-specific charts* ***(***[***Guazzi et al. 2012***](https://www.ncbi.nlm.nih.gov/pmc/articles/PMC4777325/)*) to simplify interpretation. Consistent across approaches is an emphasis on the graphical display of CPET parameters (*[***Dumitrescu and Rosenkranz 2017***](https://www.atsjournals.org/doi/10.1513/AnnalsATS.201612-955FR)*). We employ these current approaches (i.e., clusters of variables, graphical presentation of CPET data) in the present manuscript to help us and the reader understand the unique CPET presentation among individuals with ME/CFS in a rigorous way.*

*In addition, the most recent Guidelines for Exercise Testing and Prescription (11^th^ Edition) from the American College of Sports Medicine state that cardiopulmonary exercise testing with ventilatory gas exchange measurement may be particularly useful for: “differentiating cardiac versus pulmonary limitations as a cause of exertional dyspnea or impaired CRF (cardiorespiratory fitness)…”- another purpose of our study.*

*To illustrate the point that CPET affords mechanistic insight, we spent effort emphasizing our finding of reduced ventilatory efficiency or inefficient ventilation. This is an important finding, the clinical relevance of which has recently been highlighted by* ***Collins et al. 2021*** *who state, “VE/VCO2 can provide important information to evaluate the ventilatory response to exercise and help to determine underlying mechanism(s) of exercise intolerance.”). Owing to the fact that inefficient ventilation is primarily determined by the amount of CO2 produced, PaCO2, and physiological dead space (i.e., modified alveolar equation) – there are potential targets that have not been fully described in the ME/CFS literature previously. We would further emphasize that these results are novel for ME/CFS research and that detailing the cardiopulmonary responses to exercise may aid in interpreting/explaining the negative consequences of acute exercise (****Kujawki et al., 2021;*** ***Meyer et al., 2013******; ,*** [***Cook et al., 2017***](https://www.sciencedirect.com/science/article/pii/S088915911730051X)*) in ME/CFS. These data are also consistent with recent evidence that abnormal breathing patterns in those with Long-COVID and ME/CFS can be detected using CPET despite normal chest x-rays, chest CT scans, and normal resting pulmonary function tests (*[***Mancini et al., 2021***](https://www.jacc.org/doi/10.1016/j.jchf.2021.10.002)*). Collectively, the opinions of expert panels and emerging ME/CFS research highlight the utility and necessity of investigating CPET as a tool to gain mechanistic insight into exercise intolerance and inefficiency. We agree with the reviewer that additional studies are needed to extend upon the findings from this study, and we also believe these data are valuable in guiding future research aimed at more directly testing the mechanisms that might underlie the inefficient ventilatory responses that we observed.*

**References**

**Ross, R.M., 2003. ATS/ACCP statement on cardiopulmonary exercise testing. *American journal of respiratory and critical care medicine*, *167*(10), pp.1451-author.**

**Neder, J.A., Phillips, D.B., Marillier, M., Bernard, A.C., Berton, D.C. and O’Donnell, D.E., 2021. Clinical Interpretation of Cardiopulmonary Exercise Testing: Current Pitfalls and Limitations. *Frontiers in Physiology*, *12*, p.348.**

**Wasserman, K., 1997. Diagnosing cardiovascular and lung pathophysiology from exercise gas exchange. *Chest*, *112*(4), pp.1091-1101.**

**Guazzi, M., Adams, V., Conraads, V., Halle, M., Mezzani, A., Vanhees, L., Arena, R., Fletcher, G.F., Forman, D.E., Kitzman, D.W. and Lavie, C.J., 2012. EACPR/AHA Joint Scientific Statement. Clinical recommendations for cardiopulmonary exercise testing data assessment in specific patient populations.**

**Dumitrescu, D. and Rosenkranz, S., 2017. Graphical data display for clinical cardiopulmonary exercise testing. Annals of the American Thoracic Society, 14(Supplement 1), pp.S12-S21.**

**Liguori, G. and American College of Sports Medicine, 2020. *ACSM's guidelines for exercise testing and prescription*. Lippincott Williams & Wilkins.**

**Collins, S.É., Phillips, D.B., Brotto, A.R., Rampuri, Z.H. and Stickland, M.K., 2021. Ventilatory efficiency in athletes, asthma and obesity. *European Respiratory Review*, *30*(161).**

**Kujawski, S., Słomko, J., Hodges, L., Pheby, D.F., Murovska, M., Newton, J.L. and Zalewski, P., 2021. Post-Exertional Malaise May Be Related to Central Blood Pressure, Sympathetic Activity and Mental Fatigue in Chronic Fatigue Syndrome Patients. Journal of Clinical Medicine, 10(11), p.2327.**

**Meyer, J.D., Light, A.R., Shukla, S.K., Clevidence, D., Yale, S., Stegner, A.J. and Cook, D.B., 2013. Post-exertion malaise in chronic fatigue syndrome: symptoms and gene expression. Fatigue: Biomedicine, Health & Behavior, 1(4), pp.190-209.**

**Cook, D.B., Light, A.R., Light, K.C., Broderick, G., Shields, M.R., Dougherty, R.J., Meyer, J.D., VanRiper, S., Stegner, A.J., Ellingson, L.D. and Vernon, S.D., 2017. Neural consequences of post-exertion malaise in myalgic encephalomyelitis/chronic fatigue syndrome. Brain, Behavior, and Immunity, 62, pp.87-99.**

**Mancini, D.M., Brunjes, D.L., Lala, A., Trivieri, M.G., Contreras, J.P. and Natelson, B.H., 2021. Use of Cardiopulmonary Stress Testing for Patients With Unexplained Dyspnea Post–Coronavirus Disease. Heart Failure, 9(12), pp.927-937.**

**Introduction**

Lines 67: “has been” or “is”?

*Line 68: Changed to ‘is’.*

Lines 69-70: Are 12 references needed to support this statement? I know it is important to recognize other people’s work. However, this seems excessive.

*Lines 70-71: As suggested, we have reduced the number of references cited.*

Lines 74-75: What do you mean by “earlier ventilatory threshold onset”? Are you indicating a lower percent value or earlier in the strict sense of time? I am asking because the actual duration to achieve a given threshold might be not that relevant as this is protocol and fitness level dependent.

*Lines 75-80: We have clarified the phrasing here. The sentence was meant to convey lower aerobic fitness, so we are specifically referring to a lower percentage of peak VO2. We have modified the sentence to reflect this, including changing “ventilatory threshold” to “gas exchange threshold” as suggested by the reviewer. Thank you for the question.*

Line 77: Well, I guess it all depends on the characteristics of the “control” group.

*Lines 79-80: We have clarified the sentence to avoid confusion concerning control group characteristics.*

Line 88: Line 85: Again, the authors should specify what they mean by “early”.

*Thank you, we have changed this to “earlier GET.”*

Line 93: What are the authors referring to by “cardiac acceleration”? Is this the kinetics of HR and/or cardiac output?

*Line 96: This phrase reflected the terminology that was used in one of the references, however we recognize the ambiguity with this term and have rephrased for clarity. It now reads “lower HR with increasing exercise intensity compared to controls”.*

Line 101: Expressing as a percent of peak capacity has pros and cons. It should be considered that functional tasks require a given absolute metabolic rate, rather than a percent of max.

*We agree, however when the same exercise protocol is used, expressing data relative to peak aerobic capacity is useful for direct group comparisons. This method, combined with matching for aerobic fitness, provides a standardized measure of relative exercise intensity that is critical for the purposes of the present study. Moreover, measures of relative intensity (e.g., % heart rate reserve, % heart rate max, % peak oxygen consumption) are useful (although not perfect) reference points for exercise prescription.*

**Methods**

Line 139: Matching for sex and age is important. However, I think that matching for physical activity levels would have been very important. It is true that age might contribute to fitness level, but even within the same age group, activity levels will play a key role in maximal a submaximal responses to exercise testing. NOTE: after reading the full manuscript, I see that matching to fitness level was performed. This should be explained here in my view.

*Thank you for pointing this out. However, this sentence describes the recruitment for the larger MACAM study and not JUST the exercise sub-study. Although we matched participants on fitness, this was not a method used for recruitment for MCAM and thus would not be appropriate to state as such. We have clarified this in the manuscript by better delineating the “MCAM (Parent Study)” (Page 4, line 136) and “Exercise Sub-study” (Page 5, line 142) methods.*

Line 149: It should read “principal” not “principle”.

*Line 161: Thank you – change made.*

Line 157: I know that you had very limited exclusion criteria. However, smoking is certainly something that will affect performance. Additionally, should you also match groups by the number of smokers?

*Thank you for alerting us to this oversight. We have retrieved the smoking status data from the CDC and added to Table 1. There were few smokers in the study. For this dataset there were 10 total smokers, five each for ME/CFS and controls. For the fitness-matched subgroup there were 2 smokers for ME/CFS and 4 for controls. Given the small numbers, and the fact that the samples were not different in the proportion of smokers, we did not re-run our analyses.*

Lines 167-168: Technically speaking, this is not a warm-up as pedaling for 1 min against no external resistance is not enough (neither in intensity nor in duration) to produce a warm-up effect. This should be called a baseline measure. Unfortunately, although baseline measures are quite useful to stabilize the signal, a 1-min baseline is too short for that purpose. Finally, using 0 W is not the best choice as you do not really know the PO associated with baseline. I mean, the external workload is 0 W, but there is some power that needs to be generated to move the legs (which is ~15 W depending on the size of the person). This is why baseline measures often start at 20 W, so that changes in PO can be measurable.

*We apologize for our lack of clarity and incorrect description of our exercise protocol and have corrected it in the manuscript. Though unloaded cycling is common clinically for deconditioned individuals or persons with chronic lung disease, as recommended by the European Respiratory Society (ERS) Task Force (*[***Radke et al., 2019***](https://err.ersjournals.com/content/28/154/180101)*), we appreciate this choice impacts other variables that require accurate determination of power output. The exercise sites accepted this trade-off in favor of ensuring a higher probability for ME/CFS participants to achieve a maximal effort, thus selected a more clinical approach of unloaded cycling.*

*Further, the protocol delivered to each of the exercise sites stated that participants will be “sitting on the bike for a 1-minute quiet rest period” to allow the participants to “adjust to breathing on the mask”. This was followed by a “3-minute warm-up” (this was unloaded) and exercise testing began at 0 Watts and proceeded until volitional exhaustion: “0.25 watts x second gradual increase = 15 watts x 1 minute (start at 0W) where subjects will be encouraged to maintain a cadence of 60 rpms during duration of test.”*

*Upon examining the exercise tests, a large percentage of tests did not start collecting metabolic data until there was ~1 or 2 minutes of warm-up remaining. In other words, they were focused on collecting data during the exercise test and not necessarily the warm-up period. We went back reviewed each exercise test to determine how much of the warm-up period was captured (i.e., when the metabolic cart began collecting data) and whether it differed between groups. For the entire sample of participants, we measured an average of 111 (±26.4) seconds of warm-up data prior to the start of exercise. This value did not differ between groups (ME/CFS 112.64 ±23.1; Controls 109.6 ±29.5). Therefore, although not all sites collected the entire 3-minute warm-up period, we are confident that it did occur prior to starting the ramped exercise period.*

*Page 6 (lines 181-189): We have clarified the description of the protocol to more accurately reflect the tests that were conducted at the exercise sites. It now reads,* ***“Exercise testing consisted of ramped cycle ergometry to volitional exhaustion. Participants were given one-to two-minutes to acclimate to the instrumentation (i.e., breathing while wearing the facemask) while seated on the cycle ergometer. This was followed by a three-minute, unloaded warm-up. Exercise testing began at 0 Watts and work intensity was increased linearly by 5 Watts every 20 seconds (15 Watts/min) until volitional exhaustion or a point when the prescribed pedal rate could not be maintained. Participants were instructed to maintain a pedaling cadence of 60–70 revolutions per minute and were verbally encouraged to continue pedaling as long as possible.”***

*Although this does not address the 0 versus 20 Watt warm-up concern, we hope that this shows the reviewer that we did in fact begin exercise with sufficient time for achieving a stable metabolic signal and that we employed a standardized and internationally recommended exercise testing protocol that were aligned with the purpose of the research.*

***Reference***

**Radtke, T., Crook, S., Kaltsakas, G., Louvaris, Z., Berton, D., Urquhart, D.S., Kampouras, A., Rabinovich, R.A., Verges, S., Kontopidis, D. and Boyd, J., 2019. ERS statement on standardisation of cardiopulmonary exercise testing in chronic lung diseases. *European respiratory review*, *28*(154).**

Lines 179-180: Why minute 2 of the exercise? I guess it is fine because even a very unfit person should still be in the moderate intensity domain at 30 W. However, using time is tricky for this type of measurements. Also, it is odd to have a measure of lactate at peak exercise (I assume immediately after exercise cessation), but not at 1 min. By minute 3, lactate might already be going down, and the highest concentration might not have been caught at peak exercise.

*There were several considerations for our timing of lactate measurement. For the 2-minute time point, we were concerned that a significant proportion of our ME/CFS participants might stop exercise early (this was not the case) and thus we wanted to have an early lactate measure that might help us understand exercise intolerance if this were to occur. As for peak and post-exercise measurement time points, we relied upon Goodwin et al., (2007) - showing that peak lactate responses to maximal effort occur from 3-8 minutes after exercise. Thus, we measured both peak and recovery responses to capture that response if present. As can be seen in Supplemental Figure #1, mean lactate values continued to rise from peak exercise to the 3-minute recovery time point.*

***Reference***

**Goodwin, M.L., Harris, J.E., Hernández, A. and Gladden, L.B., 2007. Blood lactate measurements and analysis during exercise: a guide for clinicians. Journal of diabetes science and technology, 1(4), pp.558-569)**

Line 182: Actually, the V-Slope method defines the gas exchange threshold (GET). The ventilatory threshold (VT) requires the evaluation of ventilatory data. Additionally, if referring to the VT as synonymous of the GET, then this should be defined as VT1, as there is also the VT2, which corresponds to the respiratory compensation point (RCP). Personally, I would never determine thresholds based solely on one plot. I would rather combine plots to feel more confident in the outcome. Regardless, please use the correct terminology here and elsewhere.

*Thank you for the correction. We now use gas exchange threshold (GET) throughout the manuscript.*

Line 183: Reference #23 is not Sue et al. This worries me as it might mean that references are misplaced not only here.

*Thank you for catching this error. This was a copy-paste error. We have double checked the other references and they are correct. This was the only one that was not linked as an EndNote citation. However, the Sue reference was not included in the reference list. This has been corrected and the reference list updated accordingly.*

Lines 183-186: Using secondary criteria has limitations that have been highlighted elsewhere (PMID: 17968581). Independently of that, some of the values that you accepted are too low in my view. For example, HR >85% of predicted HRmax is a very low mark. Even RPE greater than >17 seems not too demanding for establishing maximal efforts. Also, what do you mean with “≤150 ml with an increase in work”? Based on your methods, you increased 5 W every 20 s. Are you implying every 20 s or every 1 min for this increase in VO2? Regardless, given that the increase in PO even per min is only 15 W, the expected increase in VO2 responses might be lower than 150 mL/min even if a plateau is not reached. This might be especially true towards the end of the test (PMID: 31580218). Thus, the criteria that you are proposing is quite unlikely to establish a maximal effort (which you should aim for, independently of whether you call it VO2peak or VO2max).

*Standard criteria for peak effort are essential for proper CPET interpretation and something that has hindered the ME/CFS literature. In fact, we highlighted this point in the introduction calling attention to the effect that approximately 50% of studies included in a recent meta-analysis did not consider such criteria (****Franklin et al., 2019****). In the present study, we applied rigorous standard criteria that our laboratory and others routinely employ in cycling studies. We recognize, however, that there is a debate as to which measures to apply as standard criteria for determination of peak effort, as well as what measures to apply as secondary criteria* ***(***[***Poole et al., 2008***](https://pubmed.ncbi.nlm.nih.gov/17968581/)*). This is made difficult by the fact that most criteria are derived from younger, healthier, and fit men and women – including the citation you highlighted (i.e., PMID: 17968581) – rather than sedentary, older and/or unhealthy individuals of greater relevance to our population.*

*Our choice of secondary criteria was based on considerations of both the population being studied and standardized criteria for cycling exercise. The criteria we chose are also widely used in research, endorsed by the American College of Sports Medicine, and are consistent with recommendations based on a large sample (N=861) of men and women ranging from 20-85 years of age (*[***Edvardsen et al., 2014***](https://www.ncbi.nlm.nih.gov/pmc/articles/PMC3891752/)*). This paper stated that standards for maximum criteria exhibit “great variability” and that although applying criteria that are too low can lead to underestimating VO2max, applying too strict criteria can lead to “rejection of subjects who would actually achieve a valid VO2max”. They also recommended that for the age and sex make-up of our sample that an RER of ≥ 1.10 should be used – consistent with the choice for the present study. Further, they recommended that for ages (20-49 years) that a blood lactate of ≥ 7 (mmol·L^-1^) for females and 9 mmol for males should be used, for 50-64 years >5 for females and >6 for males and for ≥65 years >3.5 for females and >4 for males. Thus, to further address the reviewers concern we went back and calculated the number of participants who also met these lactate criteria. For ME/CFS 84% of the sample met lactate criteria (in addition to the other secondary criteria). For controls, 88% met lactate criteria. We have added the peak lactate data to Table 4.*

*Finally, all participants were encouraged to provide a maximal effort to volitional exhaustion and this point was emphasized both during the instructions that were given to the subjects prior to exercise, as well as throughout the exercise test. Using multiple criteria and adjusting for age and sex when determining the values, we are confident that the included participants gave maximal efforts.*

***References***

**Franklin, J.D., Atkinson, G., Atkinson, J.M. and Batterham, A.M., 2019. Peak oxygen uptake in chronic fatigue syndrome/myalgic encephalomyelitis: A meta-analysis. International journal of sports medicine, 40(02), pp.77-87.**

**Poole, D.C., Wilkerson, D.P. and Jones, A.M., 2008. Validity of criteria for establishing maximal O 2 uptake during ramp exercise tests. European journal of applied physiology, 102(4), pp.403-410.**

**Edvardsen, E., Hem, E. and Anderssen, S.A., 2014. End criteria for reaching maximal oxygen uptake must be strict and adjusted to sex and age: a cross-sectional study. PloS one, 9(1), p.e85276.**

Line 188: I wonder how oxygen extraction can be derived from these outcomes.

*We recognize that non-invasive CPET affords estimates and cannot directly assess oxygen extraction. The latter is best characterized by arteriovenous O2 difference (a-vO2 diff) which necessitates arterial sampling. As an alternative, oxygen pulse provides some insight in this regard as it is dependent on stroke volume and a-vO2 difference. Since stroke volume plateaus during incremental protocols around mid-exercise, O2 pulse beyond this point is largely reflective of a-vO2 difference. However, this remains indirect, and your point is well-taken. Therefore, we have removed the term ‘extraction’ and replaced with ‘utilization’.*

Line 190: Based on what I discussed above with the 0 W baseline, you cannot calculate the function gain (i.e., ΔVO2/ΔWR) with any level of confidence as you do not know the actual baseline PO. Again, the external resistance was set to 0 W, but moving the legs against no external resistances required an unknown PO. Additionally, after examining the results in the graphs, the functional gain values do not make much sense. How can people be more efficient at greater intensities of exercise when the gain in the heavy and severe domains is progressively greater than that in the moderate domain? This has to do with limitations of using the ramp model to understand efficiency (PMID: 31580218).

*Thank you for catching our error. We did not calculate functional gain (ΔVO2/ΔWR), rather we reported point estimates of VO2/WR at the individual intensities as shown in Figure 1. We inadvertently included the ∆ symbol (delta) and have now revised accordingly.*

Lines 194-201: All these estimations make little sense as they are based on too many assumptions.

*We are uncertain of what assumptions the reviewer is referring to here. For CTI, there is the assumption of age-predicted max heart rate, but this is commonly accepted and applied in numerous exercise-related predictions (*[***Brubaker and Kitzman, 2011***](https://www.ahajournals.org/doi/full/10.1161/CIRCULATIONAHA.110.940577)*). Importantly, our purpose for including the chronotropic incompetence measure, as well as other indicators (e.g., percentage of heart rate reserve), was to better test whether ME/CFS was characterized by abnormal heart rate responses to exercise – something that we clearly did not observe. These data are novel and challenge the widely held notion that ME/CFS is characterized by chronotropic incompetence.*

***Reference***

**Brubaker, P.H. and Kitzman, D.W., 2011. Chronotropic incompetence: causes, consequences, and management. Circulation, 123(9), pp.1010-1020.**

**Results**

Lines 275-276: OK, this is good to know. I think you could provide with more information on this in the previous section when you refer to this point.

*Lines 267-268 We have added the matching information to the methods section as requested.*

Line 281: I think it should be “body mass” instead of “weight” (here and elsewhere, including tables).

*We are unsure what the reviewer means by this comment. We reported both weight in kg and body mass index.*

Lines 304-362: Aside from some small differences still present in the matched groups, what these data show (not surprisingly) is that fitness level modulates the responses. I would accept that someone with the condition evaluated in this study might be more likely to be less fit. However, once fitness level is matched, the condition is no longer the problem. I see that there are some minor differences still present in the groups matched by fitness level (mostly some ventilatory responses with breathing frequencies and tidal volumes), but nothing very concerning. In fact, some of the values seem very close despite the claim of significance. I guess all this will be discussed later, but I wanted to share my thoughts at this point of the process.

*We respectfully disagree. The differences that we observed were meaningful statistically (moderate effect sizes for both our simple group comparisons and during exercise) and for ME/CFS. The differences observed, after controlling for aerobic fitness, were also moderate in magnitude. Because these data are novel with respect to ME/CFS, the immediate clinical relevance for these data is unclear, but may in fact be meaningful for understanding ME/CFS and the consequences of acute exercise – i.e., the phenomenon of post-exertional malaise or symptom worsening following effort that is now part of the diagnostic criteria. In response to Reviewer 3, who asked for more information regarding clinical relevance, we have also added the following information:*

*Pages 15-16 (lines 448-459): “Moreover, determining the clinical meaningfulness of the differences observed here would require longitudinally examining whether changes in these outcomes are associated with improvements or decrements in clinically relevant outcomes (e.g., symptom severity), an approach that is not possible in this cross-sectional analysis. Further, while the convention of ≥0.5 SD is sometimes used as a metric to gauge clinical significance, it is also recommended that the practical interpretation of effect size magnitude be relativized to a particular area of study (Cohen, 1988). In the context of CPET research involving people with ME/CFS, this study is one of the largest of its kind, especially in terms of those which controlled for aerobic fitness.* *Therefore, the magnitude of the differences observed here should be viewed as reference values which future CPET studies involving people with ME/CFS can begin to make inferences about clinically meaningful changes. We acknowledge that discussion of potential mechanistic explanations for the differences observed in this study should be tempered by these considerations.”*

**Discussion**

Lines 386-389: First, I do not see the differences to be very large despite some being significant. Regardless, I accept that the authors need to discuss this, but I would tone it down a bit when referring to “important differences”. Most importantly, the statement that “those with ME/CFS are characterized by inefficient exercise ventilation suggestive of preserved oxygen delivery but poor oxygen utilization” cannot be justified by the data that the authors have presented. There is no information in this manuscript on oxygen delivery at the central or peripheral level, or anything that would imply a deficit for oxygen utilization at the mitochondrial level. Thus, these data show some differences between groups in some variables that were evaluated, but nothing that could be translated into this assertion.

*We acknowledge the limitations the reviewer has raised and have made changes to the Discussion to address the reviewers concern, particularly concerning suggestions of what mechanisms may underlie the differences in exercise responses that we identified. For this first paragraph (Page 15, lines 414-416) we have deleted the mention of “suggestive of preserved oxygen delivery but poor oxygen utilization” and now simply state that “These results show that cardiopulmonary responses to exercise among those with ME/CFS are characterized by inefficient exercise ventilation.” In addition, on* Page 17, Lines 493-495: “*Additional research into underlying mechanisms, such as mitochondrial function, are needed to further understand the observed gas-exchange inefficiencies.”*

Lines 390-412: OK, I understand that some of the differences need to be highlighted. However, the most important message is not that there were some differences in ventilatory patterns even when matching by fitness level, but rather that the matching strategy abolished the ME/CFS decline in fitness and other responses. Thus, it seems as if ME/CFS patients can have similar fitness as the control participants.

*We think that both messages are important and that this study presents novel data that can be assessed in future studies to help determine, for example, why exercise worsens ME/CFS (i.e. post-exertional malaise (PEM)), and/or determining whether improving ventilatory efficiency improves clinical aspects of the disease. We agree with the reviewer that demonstrating that fitness differences are likely explanations for many of the reported exercise differences in the ME/CFS literature, but it is too early to discount the clinical importance of our findings. Finally, we are careful to state that our results “suggest” and we in fact state that the primary limitation of the study is the “indirect nature of CPET”. We have softened the language indicting clinical meaningfulness of our data and instead have added a new paragraph to better emphasize the need for more research.*

Line 416: I am not sure I agree with the idea of a ventilatory inefficiency. In the end, the ventilatory responses adjust to respond to muscle metabolism. Thus, different metabolic conditions within the active tissues might cause the differential responses in the ME/CFS patients. It would be interesting to know more about what was happening at that level.

*We agree that knowing tissue specific responses would benefit our understanding and interpretation of the data.*

Lines 419-435: This is highly speculative. Trying to imply peripheral limitations from these data seems excessive.

*Please see our earlier response on this question.*

Lines 436-452: I still find the level of speculation too high. At least in this case, it has to do with something that was measured in this study. The first theory seems interesting as avoiding negative symptoms is something that people try to do. The second one seems less appealing to me as I am not sure there is evidence of a perfusion limitation at the alveoli level. If this was the case, would not you expect a difference in ventilatory responses even in the groups matched by fitness level?

*In this paragraph we highlighted the importance of examining the components of minute ventilation – f_R_ and V_T_ – as there was a moderate effect between groups such that individuals with ME/CFS adopted a breathing pattern that was deeper and slower than controls. A given alveolar ventilation can be achieved by different combinations of f_R_ and V_T_, and we know from chronic lung disease populations that the strategies selected are to optimize alveolar ventilation. Though we felt this interpretation reflected our data and it was appropriately acknowledged as speculation, we went ahead and deleted these sentences to address your concern.*

Lines 453-468: Again, this is just mere speculation. Similar to my previous comment, would not you expect a difference in ventilation in the groups matched by fitness level if muscle fatigue in the ME/CFS group was an issue? Perhaps I am not understanding the point the authors are making. Nevertheless, I think that the authors are trying to say far more than what they can with the data that they are presenting.

*We have deleted this paragraph from the discussion. Our hope was to stimulate future research, but we agree that this particular paragraph was a reach and not warranted given the data that we collected.*

Lines 475-485: I think that the increased RPE is an interesting finding. I was fine with the first part of this paragraph. However, in this section, the authors went back to the speculation. The reality is that the RPE is regulated by many different components as the authors indicated. However, none of the possible contributors to the RPE were measured in this study. Thus, there is not much to say other than “RPE was greater in ME/CFS but we do not know why”.

*We have reduced some of the speculation in this paragraph to focus more on ventilation. We would state that we did measure variables that have been shown to influence RPE. These include VE* ***(******Robertson et al., 1982****), lactate* ***(******Demello et al., 1987******;*** ***Robertson et al., 1986****), and force (i.e., Watts) (*[***Cafarelli, 1982***](https://europepmc.org/article/med/7154894)*) all of which when manipulated change the sense of effort during exercise. Although we did not experimentally manipulate the “normal” exercise response, we do know that these signals (VE, lactate, increasing Watts) contribute to effort sense. We have removed the speculation around Borg’s conceptions and instead have restated that RPE is consistently elevated in ME/CFS and should be considered when considering exercise recommendation (Page 17, lines 520-530).*

***References***

**Robertson, R.J., 1982. Central signals of perceived exertion during dynamic exercise. Medicine and science in sports and exercise, 14(5), pp.390-396.**

**Demello, J.J., Cureton, K.J., Boineau, R.E. and Singh, M.M., 1987. Ratings of perceived exertion at the lactate threshold in trained and untrained men and women. Med Sci Sports Exerc, 19(4), pp.354-362.**

**Robertson, R.J., Falkel, J.E., Drash, A.L., Swank, A.M., Metz, K.F., Spungen, S.A. and LeBOEUF, J.R., 1986. Effect of blood pH on peripheral and central signals of perceived exertion. Medicine and science in sports and exercise, 18(1), pp.114-122.**

**Cafarelli, E., 1982. Peripheral contributions to the perception of effort. Medicine and science in sports and exercise, 14(5), pp.382-389.**

Lines 486-513: The cardiac data are very “soft” to try to make any mechanistic implications. This paragraph is very speculative with little connection to the data that the authors presented.

*We are unsure what speculation the reviewer is referring to. We simply report here that HR responses to exercise appeared normal and present our data to support that interpretation. We describe other studies and the claims that they made, as well as the limitations of previous work, but we do not discuss the mechanistic underpinnings of ME/CFS beyond using the term chronotropic incompetence and reporting on accepted measures of these clinical indices. We have removed the reference to “sympathetic stimulation” (Page 18, line 537) in the third sentence and have removed the detailed discussion of the Montague et al. study (Page 18, lines 549-553).*

Lines 553-564: I do not see how this study can help informing practitioners on physical activity or exercise recommendations.

*We agree that this paragraph was unclear as originally written and have revised to say the following:*

*Pages 19-20 (lines 601-616): “When considering physical activity for people with ME/CFS, clinicians face the challenge of helping patients avoid the negative effects of acute exercise (e.g., symptom exacerbation), while moving them towards experiencing the health benefits associated with a more physically active lifestyle. A logical approach is to develop exercise prescriptions which strike a balance between minimizing symptom exacerbation and maximizing function, however, there is limited information on the intensity threshold at which this ideal balance occurs or guidance on how to establish this threshold for individual patients. It is noteworthy that in other patient care settings for which a substantial literature on exercise prescription already exists, ramped incremental CPET is considered the gold standard for physiologically comprehensive exercise intensity assessment and prescription. Given that over 90% of the present sample was able to provide a valid peak effort during CPET, we conclude that there is sufficient precedent for future work testing whether CPET guided exercise prescription can help address the unique physical activity challenges experienced by people with ME/CFS. Further, we believe that these data will support current recommendations to practitioners to encourage patients with ME/CFS to maintain tolerated levels of activity and to increase activity with caution and make adjustments to avoid post-exertional malaise.”*

**Reviewer #2: Thank you for submitting this manuscript for review. Please find some comments below:**

**Abstract**

Methods - Define HR on first use rather than just stating cardiac. Also include 'rating' for RPE.

*Lines 40-41: The suggested edits have been made accordingly. Thank you.*

**Intro**

- Line 80/81 - check sentence structure

*Lines 81-85: We have modified the sentence to read “Although quality of the included studies varied greatly (i.e., 16 of the 32 studies did not use criteria for determining peak effort), and the results were heterogenous, the meta-analysis reported that the mean effect difference of 5.2 ml/kg/min lower in ME/CFS compared to controls was moderate and clinically meaningful.”. We hope that this addresses the sentence structure issue.*

- Line 82 - This is not a comment per se, but more out of interest for the reader. It is up to you whether you choose to add this detail in. Was this study conducted in those with MW/CFS? Are there any contraindications of doing serial tests in this population? How does the results compare to healthy individuals?

*Line 86: Yes, this study was conducted in ME/CFS and we have made that clear in the sentence now. Thank you for the question.*

*With respect to serial testing, these studies have been conducted in this population. For example:*

- *Stevens, S., Snell, C., Stevens, J., Keller, B. and VanNess, J.M., 2018. Cardiopulmonary exercise test methodology for assessing exertion intolerance in myalgic encephalomyelitis/chronic fatigue syndrome. Frontiers in pediatrics, 6, p.242.*
- *Snell, C.R., Stevens, S.R., Davenport, T.E. and Van Ness, J.M., 2013. Discriminative validity of metabolic and workload measurements for identifying people with chronic fatigue syndrome. Physical therapy, 93(11), pp.1484-1492.*

*These studies have generally found that the “anaerobic threshold” occurs earlier on the second test and that peak exercise capacity is sometimes also reduced.* *The MCAM clinical investigators report that patients with ME/CFS who undergo repeat CPETs on consecutive days experience a significantly greater risk of illness relapse (i.e. post-exertional malaise) of greater intensity and longer duration, so there is a relative contraindication to that protocol. Although we find the topic of serial exercise testing in ME/CFS of interest, because we only report the results from single bout of exercise, we prefer to focus on what these data tell about the function of the cardiopulmonary system when someone with ME/CFS exercises. We thank the reviewer for the consideration.*

**Methods**

- Line 212 - you mention missing data here. Please explain in the statistical analysis section how you dealt with missing data points.

*Pages 7 & 8 (lines 252-254 & 277-279): We appreciate the opportunity to add this information. We have added the following to the statistical analysis section. “Missing data for group comparisons were imputed using the Multiple Imputation by Chained Equations method (REF) if ≤ 15% of the data were missing, otherwise they were handled using listwise deletion.” And “Missing data for these analyses were imputed using the Multiple Imputation by Chained Equations method (REF) if ≤ 15% of the data were missing, otherwise they were handled using pairwise deletion.”, to reflect the way in which the missing data were handled for our between group and mixed effects models, respectively.*

- Line 247 - you state here that you did not specifically match for sex. Yet earlier on in the methods (line 139) you state that they were matched on sex and age. Please can you clarify this.

*This matching occurred at the larger MCAM study-wide level and not specifically for the exercise sub-study participants reported here. The sampling procedure used by the larger MCAM study certainly aided in having a balanced male/female ratio, this was not a recruitment aspect that was specific for the exercise sub-study. We have clarified this in the manuscript by better delineating the “MCAM (Parent Study)” (Page 4, line 136) and “Exercise Sub-study” (Page 5, line 142) methods.*

- Line 159 - I understand the need for a screening ECG prior to exercise for safety. However, it seems as though you used an ECG during the tests but only measured was HR. Why was this? If this wasn't the case, explain that. If you did use ECG just for HR,

*There was a separate resting ECG study for the larger MCAM study. The ECG prep was subsequently used to determine safety for commencing exercise in this exercise study. This occurred prior to the exercise test and thus the ECG preparation was kept in place for exercise. This author (Dane Cook) was not given access to any ECG data that was saved from the exercise test – if that did occur.*

**Results**

- Tables - may look neater if there is consistency with use of decimal places. Please also ensure all acronyms are detailed in the footer. I think SBP and DBP are missing from table 1but just double check in case there are any others.

*Thank you for the suggestion. We have checked the tables and updated them to include all acronyms so that the tables can stand alone from the manuscript. As for the decimal place issue, we will consult with PLIS One and adjust the tables in accordance with their journal standards should the manuscript be accepted for publication*

**Discussion**

- Line 381 - You mention cardiac performance as you have done throughout. One question I have is, have you truly measured cardiac performance with only measuring HR? Is there maybe a better term which could be used for this? If you do change it, please ensure there is consistency of use throughout the manuscript.

*This is a good question and thank you raising as other reviewers had similar concerns. We did not investigate all measures of cardiac performance that are possible (e.g., stroke volume, ejection fraction, ECG characteristics, frequency components, etc.), but we did investigate the dynamic responses of heart rate throughout exercise and calculated clinically relevant metrics to better test for chronotropic incompetence (e.g., achievement of 85% of HRR, chronotropic incompetence index). But to the reviewer’s point, we should be more precise with the language that we use to describe our heart rate results. We have updated the manuscript throughout to remove the phrase “cardiac performance” where appropriate and replace it with more specific terminology such as “heart rate indices” or “heart rate responses”.*

**Reviewer #3: This is an interesting and important study and the manuscript is well written. I have highlighted a few methodological and statistical concerns that I would like to see addressed.**

***Thank you for the positive view of our work and for the opportunity to address your concerns.***

**ABSTRACT**

• Define ME/CFS and MCAM at first mention

*Lines 33-34 & 38: The suggested edits have been made accordingly. Thank you.*

**INTRODUCTION**

• The introduction is well written and provides a strong rationale for the study in the context of current evidence.

*Thank you. We appreciate the comment and support of our objectives.*

• Can the authors amend the study objectives to improve clarity? For example, “characterise the exercise capacity of the MCAM cohort” could be changes to something like “compare exercise capacity in patients with ME/FCS compared to healthy controls recruited from the MCAM study”.

*Page 4, lines 115-120: We have restated objectives 1 and 2 as such: “This study was conducted* *to: 1) compare exercise capacity of those with ME/CFS and otherwise healthy controls within the Multi-site Clinical Assessment of Chronic Fatigue Syndrome (MCAM) cohort, 2) to compare cardiopulmonary, metabolic, and perceptual responses to exercise in those with ME/CFS and otherwise healthy controls, and 3) determine the role of aerobic fitness on the exercise response, efficiency, and HR variables of interest.*

**METHODS**

Recruitment:

• Please list the major inclusion and exclusion criteria used to identify eligible participants for the MCAM study (this allows readers to read this paper without having to read the MCSM study paper too).

*Page 5, lines 144-150: We have added the following information to the methods section: “In brief, medical records were reviewed by clinic staff or study coordinators and screening was conducted by telephone. ME/CFS participants were excluded from the study if illness onset occurred after 62 years of age, they had human immunodeficiency virus infection, were currently pregnancy, or had dementia, or the participants could not read English at an eighth-grade level. Healthy control participants could not be younger than 18 or older than 70 years of age, self-reported good health, had no history of ME/CFS, and no other active illnesses.”*

Pre-exercise testing:

• What objective criteria, based on HR, ECG morphology and metabolic responses, were used to “ensure it was safe to initiate exercise testing”?

*Thank you for this question. According to the MCAM protocol, “At the end of the rest interval an automatic heart ECG recording will be collected to observably quantify the resting heart rate at the time of the test and to determine if there are any pre-existing cardiac conditions that may exist that may or may not preclude the subject from the maximal testing but collection of which may be later used for correlation type of analysis.” Although ECG data were obtained and reviewed prior to exercise testing, we did not have access to any of the clinical interpretation beyond that each participant in this study was deemed safe for exercise (as stated in the Methods) from both their physician and the initial resting ECG.*

Exercise testing:

• Can the authors explain and justify why an individually-calculated work rate increment wasn’t used? The work rate increment should be set so that CPET lasts 8-12 minutes. Using the same work rate increment for a group of people with heterogenous fitness levels will lead to different CPET durations, which may influence peak VO2.

*We agree that individualized work rate increments are best practice per AHA guidelines. This is particularly important for clinical populations whereby AHA guidelines state (*[***Fletcher et al., 2013***](https://www.ahajournals.org/doi/full/10.1161/CIR.0b013e31829b5b44)*): “If testing is performed, protocols that begin at low work rates and have low work rate increments per stage, including individualized ramp protocols, can be especially useful.” It is the low work rate increments that are most critical; hence our 5 Watt increment every 20-seconds protocol. Given the multi-site nature of the study – standardization and consistency in the execution of CPET across sites is not trivial. Therefore, a low work rate increment protocol is preferable.*

*While it is true that an individualized work rate protocol may yield ‘significantly’ greater total exercise duration and/or peak VO2, the clinical relevance of this difference is less well understood. Additionally, the impact of incremental work rate steps – when low – may have even less impact on CPET parameters of interest to the present study as demonstrated in a small clinical sample of patients with pulmonary hypertension* ***(***[***Glaser et al., 2008***](https://link.springer.com/article/10.1186/1471-2466-8-3)*).*

*We also agree that heterogeneous fitness levels could potentially influence CPET duration - which further underscores our decision to focus on the analysis whereby groups were matched on fitness. Due to matching, we have greater confidence that the observed between-group differences, despite similar VȮ_2_, are clinically meaningful.*

*Still, we appreciate the Reviewer’s concern in keeping with recommended best practices. To assuage this concern, we performed a post-hoc calculation of individualized work rate increment using both estimated and observed VȮ_2_ for the calculation of work rate increments. We found the predicted and observed work rate increments were very similar between groups as demonstrated in the table below, and also quite similar to our 20 W·min^-1^ protocol.*

|  | *ME/CFS* | *Controls* |
| --- | --- | --- |
| *Predicted VȮ_2_* | *15.2 ± 6.7* | *14.6 ± 7.3* |
| *Observed VȮ_2_* | *15.1 ± 6.9* | *15.6 ± 6.3* |

***References:***

**Fletcher, G.F., Ades, P.A., Kligfield, P., Arena, R., Balady, G.J., Bittner, V.A., Coke, L.A., Fleg, J.L., Forman, D.E., Gerber, T.C. and Gulati, M., 2013. Exercise standards for testing and training: a scientific statement from the American Heart Association. Circulation, 128(8), pp.873-934.**

**Gläser, S., Lodziewski, S., Koch, B., Opitz, C.F., Völzke, H. and Ewert, R., 2008. Influence of the incremental step size in work rate on exercise response and gas exchange in patients with pulmonary hypertension. BMC pulmonary medicine, 8(1), pp.1-6.**

• Can the authors explain and justify why the warm-up only lasted 1-minute? It is reasonably well-established that ~3 minutes is required to reach a steady state for hearty rate and ventilation (which is the main point of the warm-up) and that is reflected in CPET guidelines (e.g. doi: 10.1016/j.bja.2017.10.020).

*Thank you for this comment and the opportunity to clarify our exercise protocol. As we described to reviewer 1, who had a similar concern, we did not accurately describe the protocol (See below).*

*The protocol delivered to each of the exercise sites stated that participants will be “sitting on the bike for a 1-minute quiet rest period” to allow the participants to “adjust to breathing on the mask”. This was followed by a “3-minute warm-up” (this was unloaded) and exercise testing began at 0 Watts and proceeded until volitional exhaustion: “0.25 watts x second gradual increase = 15 watts x 1 minute (start at 0W) where subjects will be encouraged to maintain a cadence of 60 rpms during duration of test.”*

*Upon examining the exercise tests, a large percentage of tests did not start collecting metabolic data until there was ~1 or 2 minutes of warm-up remaining. In other words, they were focused on collecting data during the exercise test and not necessarily the warm-up period (although a 3-minute warm-up period did occur). We went back reviewed each exercise test to determine how much of the warm-up period was captured and whether it differed between groups. For the entire sample of participants, we collected an average of 111 (±26.4) seconds of warm-up data prior to the start of exercise. This value did not differ between groups (ME/CFS 112.64 ±23.1; Controls 109.6 ±29.5).*

*Page 6 (lines 180-188): We have clarified the description of the protocol to more accurately reflect the tests that were conducted at the exercise sites. It now reads,* ***“Exercise testing consisted of ramped cycle ergometry to volitional exhaustion. Participants were given one-to two-minutes to acclimate to the instrumentation (i.e., breathing while wearing the facemask) while seated on the cycle ergometer. This was followed by a three-minute, unloaded warm-up. Exercise testing began at 0 Watts and work intensity was increased linearly by 5 Watts every 20 seconds (15 Watts/min) until volitional exhaustion or a point when the prescribed pedal rate could not be maintained. Participants were instructed to maintain a pedaling cadence of 60–70 revolutions per minute and were verbally encouraged to continue pedaling as long as possible.”***

•More details are required in the determination of the ventilatory threshold. Were ventilatory equivalents and/or end-tidal pressures used to confirm the ventilatory threshold alongside the V-slope method? How many investigators independently assessed the ventilatory threshold, and if so, how were discrepancies resolved? Were there any instances where the ventilatory threshold could not be ascertained?

*Page 6 (lines 198-200): Thank you for this question. We did not assess ventilatory equivalents or other gas exchange threshold measures in addition to the V-slope method. The V-slope method was independently assessed by two investigators. Discrepancies were resolved by a third assessor (the first author). This information has been added to the Methods. There were six cases (all ME/CFS) where a V-slope could not be derived.*

*To address your concern, we analyzed a subset of our sample (n = 109) using the ventilatory equivalents method in comparison to our V-slope approach using the same 2 independent investigators who performed original analyses. Overall, we found strong agreement between approaches. Specifically, the GET occurred at a similar percentage of peak VO2 (V-slope: 53.1%; Ventilatory equivalents: 55.7%). Given the similarities and focus of the present manuscript, we have decided to retain our original analyses. Moreover, as we focus our analysis and interpretation primarily on the entire exercise response (i.e., relative intensity plots) – we believe this decision is justified.*

• How were data artifacts in the raw CPET data objectively defined? I.e. what constituted a data artifact?

*Data artifacts were defined as those points where “clearly non-physiological values (e.g., heart rate = 300 bpm at a single breath surrounded by heart rates of 80 bpm at the beginning of exercise) and that would influence the 20-sec averages. These points were deleted from the cleaned test. It is important to note that these occurrences were very rare and were included for transparency. We will gladly add the information to the manuscript if the reviewer deems it critical.*

• For future reference, the authors may wish to consider averaging the data using moving average of the middle five of seven breaths. This would automatically remove [most, if not all] data artefacts.

*Thank you for this suggestion. We will certainly consider it for future studies.*

Statistical analyses

• Can the authors make the raw data available? This would only include anonymysed CPET data (no personal information). This aligns with PLOS ONE’s policy on data availability: “Authors are required to make all data underlying the findings described fully available, without restriction, and from the time of publication”

*Because we contracted with the CDC, this author (the PI Dane B. Cook) cannot make the data publicly available. However, we made the CDC aware of this request and they sent the following language based on a previous paper that they published in PLOS One:*

*“Restrictions by the data custodians mean that the datasets are not publicly available or able to be provided by the authors. Researchers wanting to access the datasets used in this study should email CDC’s ME/CFS Program (cfs@cdc.gov) and discuss next steps for the data request. The ME/CFS program data review committee will grant the access after the review and the data use agreement is finalized. “*

*We have added this information in the acknowledgments section of the manuscript. We thank the reviewer for this request as it promotes data transparency.*

• Can the authors also make the SPSS syntax available? Or at least a snippet/example of the SPSS syntax used? This would allow peer reviewers and readers to reproduce the results and check the veracity of the findings.

*As referred to above, the CDC has informed us that this information can be requested along with the request for data.*

• To my knowledge, SPSS does not report Hedges g (admittedly I haven’t used SPSS for a long time). If this is the case, how were the Hedges g and corresponding 95% confidence intervals calculated?

*Hedges g were not calculated within SPSS. We calculated it separately within Excel.*

• Furthermore, Hedges g can be calculated in a number of different ways (see https://www.frontiersin.org/articles/10.3389/fpsyg.2013.00863/full). Therefore, please could the authors report the formula they used to calculate Hedges g?

*We used the following formula within excel for determining Hedges g:*

**Hedges’ g =** $\frac{\boldsymbol{M}\boldsymbol{1} \boldsymbol{-M}\boldsymbol{2}}{\boldsymbol{S}_{\boldsymbol{p}}}$

- **M_1_ = mean of group 1**
- **M_2_ = mean of group 2**
- **S_p_ = pooled standard deviation**

**S_p_ =** $\boldsymbol{\surd}\frac{\left( \boldsymbol{n}_{\boldsymbol{1}}\boldsymbol{-1} \right){\boldsymbol{S}_{\boldsymbol{1}}}^{\boldsymbol{2}}\boldsymbol{+}\left( \boldsymbol{n}_{\boldsymbol{2}}\boldsymbol{-1} \right){\boldsymbol{S}_{\boldsymbol{2}}}^{\boldsymbol{2}}}{\left( \boldsymbol{n}_{\boldsymbol{1}}\boldsymbol{-1} \right)\boldsymbol{+}\left( \boldsymbol{n}_{\boldsymbol{2}}\boldsymbol{-1} \right)}$

- **n_1_ = sample size of group 1**
- **S_1_ = standard deviation of group 1**
- **n_2_ = sample size of group 2**
- **S_2_ = standard deviation of group 2**

**Small sample size correction =** $\left( \boldsymbol{1-}\frac{\boldsymbol{3}}{\boldsymbol{4}\boldsymbol{N-9}} \right)\boldsymbol{*g}$

- **N = total sample size**
- **g = Hedges’ *g***

• The authors report that sensitivity analyses (i.e. excluding participants taking cardiovascular acting drugs) did not substantially change the results. However, the actual results are not presented. Please can the authors report the findings of all sensitivity analyses as supplementary information.

*We have included these analyses within the supplemental materials as requested. We thank the reviewer for this suggestion.*

• In the mixed models, were the random effects intercept-only or did they incorporate random slopes?

Page 8 (lines 261-262): We included random effects for the Intercept only. We have added this information to the Methods section: “Fixed *Effects included Group, Time, Age, and Group*Time and the intercept was included as a Random Effect.”*

**RESULTS**

• Please give reasons why the 4 participants were withdrawn from the study, or state that the reasons are unknown.

*Page 8 (line 291): Participants were not asked why they withdrew, so we have added “reasons unknown” to the manuscript.*

• Did participants self-report whether they adhered to the pre-testing restrictions? (No smoking for 2 hours, caffeine for 4 hours etc).

*Page 5 (lines 169-171): Yes, per the study protocol, each study site inquired about the pre-testing restriction to ensure that participants had adhered. We have added this information to the Methods. It reads: “Participants were instructed to abstain from smoking for 2 hours, ingesting caffeine or food for 4 hours, and exercising for 24 hours before testing. Compliance with these instructions were confirmed via self-report of the participant prior to testing.”*

• Please report CPET duration for both groups.

*These data have been added to Table 4. Thank you for catching this omission.*

• Please report comparisons in the original units of measurements, as well as Hedges g. This could be reported as supplementary information if needed. The difference in original units is often more informative than standardised effect sizes because it can be judged against the MCID or measurement error.

*Thank you for the suggestion. We have included tables of the original units as suggested as supplementary data.*

• The SD for ventilatory threshold as a % of peak VO2 is implausibly tiny (0.1%). I would expect substantially higher variability. Could the authors double check their data and code/syntax? In my opinion, this is an example why making data and code publicly available is so useful.

*We agree that making data publicly available is important and we thank the reviewer for spotting the data error. We checked and the error was in the placement of the decimal since our %percent peak VO2 data output listed the data as fraction of 1 rather than as a % of 100 (e.g., .529 ±0.11). We checked the other measures in Table 2 and they are accurate.*

**DISCUSSION**

• Can the authors elucidate on the clinical meaningfulness of some their differences? For example, do the authors think the 6.5 ml/kg/min difference exceeds the MCID or measurement error?

*Thank you for this important question. We are unaware of MCIDs for CPET parameters in adults with ME/CFS using either anchor- distribution- or qualitative-based methods. Davenport et al. 2020 (*[*Davenport et al., 2020*](https://content.iospress.com/articles/work/wor203170)*) computed smallest real difference (SRDs) derived from 51 adults who performed two consecutive CPETs. They observed the SRD for relative VO2 was 5.1 ml/kg/min, and we have now added this point to the discussion. With respect to our other observed differences – i.e., fR, VT, VE/VCO2 and VE/VCO2 – these parameters were not described by Davenport and colleagues. While the SRD is important to identify change above and beyond measurement error, it does not tell us anything about clinically important differences. Much in the same way, it is important to acknowledge that while Cohen’s rules of thumb of 0.2, 0.5, and 0.8 provide a helpful starting point for interpreting effect size magnitude, he also recognized that what would be a small, medium, and large effect size would in practice depend on the particular area of study (Cohen, 1988). In terms of adjusting CPET findings for aerobic fitness, this is the largest study of its kind. Thus, consistent with Cohen’s (1988) suggestion, we believe that the values observed here should be viewed as a benchmark which future studies can use to make inferences about clinical meaningfulness and that discussion of the clinical meaningfulness of our results may be preliminary. In an effort to make our position clearer, we have made the following edit to the discussion.*

*Pages 15-16 (lines 448-459): “Moreover, determining the clinical meaningfulness of the differences observed here would require longitudinally examining whether changes in these outcomes are associated with improvements or decrements in clinically relevant outcomes (e.g., symptom severity), an approach that is not possible in this cross-sectional analysis. Further, while the convention of ≥0.5 SD is sometimes used as a metric to gauge clinical significance, it is also recommended that the practical interpretation of effect size magnitude be relativized to a particular area of study (Cohen, 1988). In the context of CPET research involving people with ME/CFS, this study is one of the largest of its kind, especially in terms of those which controlled for aerobic fitness. Therefore, the magnitude of the differences observed here should be viewed as reference values which future CPET studies involving people with ME/CFS can begin to make inferences about clinically meaningful changes. We acknowledge that discussion of potential mechanistic explanations for the differences observed in this study should be tempered by these considerations.”*

***References:***

**Davenport, T.E., Stevens, S.R., Stevens, J., Snell, C.R. and Van Ness, J.M., 2020. Properties of measurements obtained during cardiopulmonary exercise testing in individuals with Myalgic Encephalomyelitis/Chronic Fatigue Syndrome. Work, 66(2), pp.247-256.**

**Cohen, J., 2013. Statistical power analysis for the behavioral sciences. Academic press.**

• Please refrain from using the term “exercise efficiency” and use “ventilatory efficiency” instead. Exercise efficiency could be confused with a measure of mechanical efficiency.

*We agree and thank you for pointing this out. All mention of ‘exercise efficiency’ has now been changed to ‘ventilatory efficiency’ or revised.*

• The CPET durations are reported in the discussion – please report these in the results section (as noted above). Furthermore, please perform a test to compare these values. There appears to be a difference in duration (9 vs 12 mins), which is most likely due to the inadequate individualisation of ramp rate, which is a major limitation of the data.

*Thank you for pointing out this omission. We have reported the exercise durations in the results section (Table 4) as requested. To the reviewer’s point, our durations were 658 (±182) seconds for the ME/CFS group and 741 (±211) for controls. These are approximately 11 and 12 minutes, respectively. The differences were moderately (effect size = ~.4) and significantly different, but within the expected range for this max test protocol. Further, the duration differences were due to the fitness differences between the groups, as we expected to find. When we compared the durations for the fitness matched groups, these differences were eliminated (p>0.05, effect size = .03) with mean exercise durations of 679.8 (±192) for ME/CFS and 673.1 (±190) for controls.*

• As above, please note the inadequate warm-up (1 min not sufficient to reach steady state) and one-size-fits-all ramp rate as limitations to the study that may have influenced oxygen kinetics.

*Please refer to our explanation of the warm-up concern above. We have also added non-individualized ramp rate as a potential limitation as suggested by the reviewer. In the limitation section, we state that:*

*Page 18 (lines 563-568): “The choice of a single ramp rate for all participants resulted a range of exercise durations and differences based on fitness. However, exercise duration differences were eliminated for the fitness-matched subgroup. Future research employing individualized work-rate increments will be important towards replicating and extending the present findings and further determining aerobic fitness in ME/CFS. To our knowledge, these studies have not been conducted.”*

I hope you find my comments helpful. Feel free to email me if you would like to discuss any of them.

*We found your comments to be very helpful and appreciate the time and effort. We think that the manuscript has been significantly improved because of them. Thank you.*

Sam Orange

sam.orange@newcastle.ac.uk
