# Supplementary material for: Cardiopulmonary, metabolic, and perceptual responses during exercise in Myalgic Encephalomyelitis/Chronic Fatigue Syndrome (ME/CFS): A Multi-site Clinical Assessment of ME/CFS (MCAM) sub-study
Source: PLoS One. 2022 Mar 15;17(3):e0265315. doi: 10.1371/journal.pone.0265315 (PMC8923458; doi:10.1371/journal.pone.0265315)
Supplement: Attachment — Submitted filename: Response to Reviewers 2.docx [file pone.0265315.s007.docx]

**We would once again like to thank the reviewers for their thoughtful review of our work. Please find our point-by-point response below.**

**Reviewer #1: Reviewer #1: I would like to thank the authors for addressing my comments. This is tricky review for me to complete. On the one hand, there are components of the manuscript that are fundamentally flawed. In many occasions, the authors justify their approaches by saying that "experts" or society guidelines endorse what they did. However, something that is conceptually flawed remains wrong even if there are pieces of the literature that would help justifying it. I could explain my position in detail but it would be pointless at this stage. On the other hand, the authors have made substantial changes to the manuscript and the other reviewers seemed pretty happy with the overall content. Then, I feel that it is not fair to engage in further discussion on some points that I think remain contentious but that cannot be changed anyway. Thus, I thank again the authors for their responses and I will not provide any further comments as I do not think there is any further meaningful contribution that I can make to the process.**

**We thank the reviewer for their efforts and contributions towards improving our work.**

**Reviewer #2: (No Response)**

**We thank the reviewer for their efforts and contributions towards improving our work.**

**Reviewer #3: Thank you for addressing the majority of my comments. Please review a couple of additional minor comments below.**

**#1 More details are required in the determination of the ventilatory threshold. Were ventilatory equivalents and/or end-tidal pressures used to confirm the ventilatory threshold alongside the V-slope method? How many investigators independently assessed the ventilatory threshold, and if so, how were discrepancies resolved? Were there any instances where the ventilatory threshold could not be ascertained?**

**Page 6 (lines 198-200): Thank you for this question. We did not assess ventilatory equivalents or other gas exchange threshold measures in addition to the V-slope method. The V-slope method was independently assessed by two investigators. Discrepancies were resolved by a third assessor (the first author). This information has been added to the Methods. There were six cases (all ME/CFS) where a V-slope could not be derived.**

**To address your concern, we analyzed a subset of our sample (n = 109) using the ventilatory equivalents method in comparison to our V-slope approach using the same 2 independent investigators who performed original analyses. Overall, we found strong agreement between approaches. Specifically, the GET occurred at a similar percentage of peak VO2 (V-slope: 53.1%; Ventilatory equivalents: 55.7%). Given the similarities and focus of the present manuscript, we have decided to retain our original analyses. Moreover, as we focus our analysis and interpretation primarily on the entire exercise response (i.e., relative intensity plots) – we believe this decision is justified.**

**Reviewer response: Thank you for addressing my comment. However, I think there was some confusion. I did not suggest that ventilatory equivalents should be used instead of the V-slope method to determine the ventilatory threshold – they should be used together, in line with guidelines (https://doi.org/10.1016/j.bja.2017.10.020). In other words, for future reference, ventilatory equivalents should be used to help confirm the ventilatory threshold determined by the V-slope method.**

**Please confirm how discrepancies were objectively defined. I.e. what counted as a discrepancy? For example, we have previously used a disagreement of >7.5% to objectively define a discrepancy. Please clarify here and add this information to the methods.**

**Thank you for this clarification and we apologize for the confusion. And thank you for the link to the excellent resource. We will be certain to combine methods for future studies.**

**We have added the following information to the manuscript describing how we objectively defined discrepancies for ventilatory threshold determination:**

**“For each subject, breath-by-breath VCȮ2 was plotted against VȮ2 to visually identify the tangential breakpoint in the VCȮ2-VȮ2 relationship. A 20-sec average around this point (10 sec before and after) denoted the ventilatory anaerobic threshold. Inter-rater differences in CPET parameters (i.e., non-identical 20 second averages) at the time of the ventilatory anaerobic threshold were flagged and adjudicated by the supervising investigator (DBC).”**

**In other words, if the 20 sec averages did not match for any of the CPET variables, the differences were examined, and a final decision was made by me.**

**# 2 To my knowledge, SPSS does not report Hedges g (admittedly I haven’t used SPSS for a long time). If this is the case, how were the Hedges g and corresponding 95% confidence intervals calculated?**

**Hedges g were not calculated within SPSS. We calculated it separately within Excel.**

**Reviewer response: This is confusing for readers because the statistical analysis section literally states in the first line that the statistical analyses were conducted in SPSS. Please add something along the lines of: “Standardised effect sizes were calculated in Microsoft Excel as the mean difference between groups divided by the pooled SD, with a Hedges g correction applied to adjust for sample bias.”**

**I have also just noticed that you have referred to “Hedges’ d” – this does not exist. Please change to “Hedges’ g” throughout the manuscript.**

**Thank you for alerting us to this. We have made the following changes to the manuscript to more accurately reflect our analytic methods.**

**“Statistical analyses were conducted using SPSS for Windows (version 26.0.1; SPSS, Chicago, IL) with the exception of the standardized effect size calculations which were calculated using Microsoft Excel as the mean difference between groups divided by the pooled SD, with a Hedges g correction applied to adjust for sample bias.”**

**We have also replaced any reference to Hedges *d* with Hedges *g*.**
